# Estimation of Eating Disorders Prevalence by Age and Associations With Mortality in a Simulated Nationally Representative US Cohort

**DOI:** 10.1001/jamanetworkopen.2019.12925

**Published:** 2019-10-09

**Authors:** Zachary J. Ward, Patricia Rodriguez, Davene R. Wright, S. Bryn Austin, Michael W. Long

**Affiliations:** 1 Center for Health Decision Science, Harvard T.H. Chan School of Public Health, Boston, Massachusetts; 2 Comparative Health Outcomes, Policy, and Economics Institute, University of Washington, Seattle; 3 Department of Pediatrics, University of Washington School of Medicine, Seattle; 4 Department of Social and Behavioral Sciences, Harvard T.H. Chan School of Public Health, Boston, Massachusetts; 5 Division of Adolescent and Young Adult Medicine, Boston Children’s Hospital, Boston, Massachusetts; 6 Department of Prevention and Community Health, Milken Institute School of Public Health, George Washington University, Washington, DC

## Abstract

**Importance:**

Eating disorders (EDs) are common psychiatric disorders associated with high mortality. However, data on ED disease dynamics and treatment coverage are sparse.

**Objectives:**

To model the individual-level disease dynamics of ED from birth to age 40 years and to estimate the association of increased treatment coverage with ED-related mortality.

**Design, Setting, and Participants:**

In this decision analytical model study, an individual-level Markov state transition model was empirically calibrated in April 2019 using a Bayesian approach to synthesize available clinical and epidemiologic ED data. The simulation model was calibrated to nationally representative US survey data from 2007 and 2011. A virtual cohort of 100 000 individuals (50 000 [50%] male) was modeled from birth to age 40 years for 4 ED diagnoses: anorexia nervosa, bulimia nervosa, binge eating disorder, and other specified feeding and eating disorders.

**Exposures:**

Age-specific ED incidence and mortality rates and background (all-cause) mortality.

**Main Outcomes and Measures:**

The main outcomes were age-specific 12-month and lifetime ED prevalence and number of deaths per 100 000 general population individuals by age 40 years. The mean and 95% uncertainty intervals (UIs) of 1000 simulations, accounting for stochastic and parameter uncertainty, are reported.

**Results:**

The highest estimated mean annual prevalence of ED occurred at approximately age 21 years for both male individuals (7.4%; 95% UI, 3.5%-11.5%) and female individuals (10.3%; 95% UI, 7.0%-14.2%), with lifetime mean prevalence estimates increasing to 14.3% (95% UI, 9.7%-19.0%) for male individuals and 19.7% (95% UI, 15.8%-23.9%) for female individuals by age 40 years. Ninety-five percent of first-time cases occurred by age 25 years. Current treatment coverage averts an estimated mean of 41.7 deaths per 100 000 people (95% UI, 13.0-82.0 deaths per 100 000 people) by age 40 years, whereas increasing treatment coverage for all patients with ED could avert an estimated mean of 70.5 deaths per 100 000 people by age 40 years (95% UI, 26.0-143.0 deaths per 100 000 people).

**Conclusions and Relevance:**

In this simulation modeling study, the estimated lifetime prevalence of ED was high, with approximately 1 in 7 male and 1 in 5 female individuals having an ED by age 40 years. The initial onset of EDs was highly concentrated during adolescence and young adulthood, suggesting that this is a critical period for prevention efforts. However, the high estimated prevalence of recurring ED later in life highlights the importance of identification and treatment of ED at older ages as well. These findings suggest that increasing treatment coverage could substantially reduce ED-related mortality.

## Key Points

**Question:**

What is the association between mortality and increasing treatment coverage for eating disorders, taking into account individual-level eating disorder disease dynamics?

**Findings:**

In this decision analytical model study, the estimated lifetime prevalence of eating disorders is high, with nearly 1 in 7 male individuals (14.3%) and 1 in 5 female individuals (19.7%) experiencing an eating disorder by age 40 years. Increasing treatment coverage for all eating disorder cases could avert 70.5 deaths per 100 000 individuals by age 40 years.

**Meaning:**

The findings suggest that increasing treatment coverage for eating disorders could substantially reduce the mortality associated with these common psychiatric disorders.

## Teaser

This decision analytical model study uses a virtual cohort of 100 000 individuals modeled from birth to age 40 years for 4 eating disorder diagnoses to examine whether increasing treatment coverage could reduce the mortality associated with eating disorders.

## Introduction

Eating disorders (EDs), including anorexia nervosa (AN), bulimia nervosa (BN), binge eating disorder (BED), and other specified feeding and eating disorders (OSFED), are common psychiatric disorders in the United States, especially among adolescents,^1^ and are associated with lower quality of life, increased health care utilization and costs, and high mortality rates.^2,3^ However, the lack of data on ED dynamics across the life course is a major barrier to developing evidence-based strategies for comprehensive ED treatment and prevention. Although some cross-sectional measures of ED prevalence are available,^1,4^ longitudinal estimates of ED dynamics, such as incidence, remission, and treatment response, are limited. In addition, population-level prevalence estimates can mask substantial heterogeneity of individual patient histories. A better understanding of ED dynamics over an individual’s life course can, therefore, help decision-makers and clinicians better target policies and programs.

However, long-term longitudinal studies that track individual-level ED histories across the life course are likely infeasible and, even if they were undertaken, would take many years to provide information. In the meantime, policy and clinical decisions must be made on the basis of the current evidence. To that end, we developed a microsimulation (ie, individual-level) model to synthesize the best available epidemiologic and clinical ED data in an internally consistent framework. By combining aggregate data on period prevalence and cumulative prevalence at different ages, we constructed and fitted a model of individual-level ED histories consistent with population-level epidemiologic data, which offers insight into person-level events and transitions. The aim of this study is to model the disease dynamics of ED from birth to age 40 years, accounting for heterogeneity, and to estimate the association of ED mortality with increasing treatment coverage.

## Methods

### Overview

We developed the model structure and set initial bounds for the parameters on the basis of epidemiologic and clinical data from the literature. We used a Bayesian framework to fit the model, where the model parameters are considered random variables. We then calibrated the model so that our simulated estimates were consistent with estimates of age-specific 12-month and lifetime ED prevalence from nationally representative surveys. We used the calibrated model to estimate the association of increasing treatment and prevention with ED mortality, accounting for heterogeneity in disease history from birth to age 40 years.

This study was deemed exempt from review and the need for informed consent by the Boston Children’s Hospital institutional review board because it is a simulation-based study and not human subjects research. This study follows the Consolidated Health Economic Evaluation Reporting Standards (CHEERS) reporting guideline for model-based analyses.

### Model Structure

We developed an individual-level Markov state transition model with 6 states: healthy, AN, BN, BED, OSFED, and deceased. These ED states are based on the *Diagnostic and Statistical Manual of Mental Disorders*,* Fifth Edition *(*DSM-5*).^5^ We assume that OSFED includes cases of subthreshold AN, BN, and BED. Because definitions of EDs have evolved over time, our modeled BED and OSFED states comprise a substantial portion of ED cases previously referred to as “eating disorder not otherwise specified” (EDNOS) in earlier versions of the *DSM*. We did not include the new *DSM-5* diagnosis “avoidant restrictive food intake disorder” (ARFID) as a separate state because of the lack of historical data.

Transitions among all states were allowed, with the exception of deceased, which is an absorbing state. We modeled transitions using an annual model cycle and followed a simulated cohort of 100 000 individuals (50% male and 50% female) from birth until age 40 years (eAppendix 1 in the Supplement).

### Model Parameters

For each model parameter, we used the best available data to set prior probability distributions to inform the calibration search bounds. To better explore the parameter space, we used uniform prior distributions, with the prior mean informed by available data (ie, empirical Bayes approach). All parameters are sex specific to account for potential differences in ED dynamics by sex. We describe the sources of data^6,7,8,9,10,11,12,13,14,15,16^ and the prior search bounds for each parameter in this section. See the Table for a summary and eAppendix 2 and eFigure 1 in the Supplement for full details.

**Table.** Model Parameter Search Bounds and Calibrated Values

Parameter	Prior Search Bounds, Uniform Distribution	Calibrated Values, Mean (95% UI)	Source
Naive incidence^a^			
AN	Age- and sex-specific	NA^b^	Global Burden of Disease Study 2017^6^
BED	Age- and sex-specific	NA^b^	Stice et al^7^ report 8-y cumulative incidence of 2.6% for female patients; age-specific estimates were imputed according to Global Burden of Disease Study 2017^6^ curves
BN	Age- and sex-specific	NA^b^	Global Burden of Disease Study 2017^6^
OSFED	Age- and sex-specific	NA^b^	Stice et al^7^ report 8-y cumulative incidence of 24.2% for female patients; age-specific estimates were imputed according to Global Burden of Disease Study 2017^6^ curves
First-year relapse probability, %^a^			
AN	5-40	Male: 21.3 (2.9-52.7)	Berends et al^11^ report overall relapse rate of 31%
		Female: 20.4 (2.3-55.6)	
BED	5-40	Male: 9.8 (0.7-28.2)	Hudson et al^10^ report relapse rate of 32.1% within 6 mo
		Female: 25.2 (3.6-52.0)	
BN	5-40	Male: 25.0 (2.2-56.9)	Olmsted et al^8^ report relapse rate of 31% at 2 y; Olmsted et al^9^ report relapse rate of 27.6% within 6 mo of treatment completion
		Female: 17.7 (1.4-47.0)	
OSFED	5-40	Male: 12.2 (0.1-50.6)	Stice et al^7^ report relapse rate of 20%-30% for EDNOS subtypes
		Female: 13.1 (0.1-52.1)	
Relapse decay parameter^a^			
AN	0-1	Male: 0.52 (0.07-0.94)	Uniform prior
		Female: 0.52 (0.05-0.94)	
BED	0-1	Male: 0.43 (0.01-0.95)	
		Female: 0.57 (0.07-0.95)	
BN	0-1	Male: 0.49 (0.03-0.97)	
		Female: 0.49 (0.03-0.89)	
OSFED	0-1	Male: 0.49 (0.02-0.93)	
		Female: 0.42 (0.05-0.90)	
Annual probability of remission without treatment, %^a^			
AN	0-10	Male: 7.8 (0.2-20.1)	Bergh et al^12^ and Hay et al^13^; based on control groups in trial
		Female: 8.5 (0.9-20.3)	
BED	0-10	Male: 16.3 (6.4-27.3)	
		Female: 7.0 (0.3-21.5)	
BN	0-10	Male: 8.5 (1.4-18.9)	
		Female: 10.0 (2.1-21.7)	
OSFED	0-10	Male: 10.9 (1.6-24.6)	
		Female: 13.6 (3.6-27.2)	
Treatment proportion, %^a^			
AN	Male: 0-50	Male: 28.2 (2.4-49.0)	Hudson et al^4^; assumed annual proportion is 50% of lifetime
	Female: 0-30	Female: 14.0 (1.9-28.9)	
BED	Male: 0-51	Male: 30.1 (4.8-50.2)	Hudson et al^4^; prior search bounds based on 95% CI
	Female: 10-53	Female: 29.7 (11.0-52.2)	
BN	Male: 0-30	Male: 15.7 (1.3-28.5)	Hudson et al^4^; for male patients, assumed annual proportion is 50% of lifetime, and for female patients, the proportion is based on 95% CI
	Female: 0-37	Female: 17.7 (1.9-35.8)	
OSFED	Male: 0-30	Male: 16.5 (2.1-28.5)	Same prior search bounds as for AN used
	Female: 0-30	Female: 16.7 (1.4-28.8)	
Treatment efficacy, relative rate of remission^a^			
AN	1.5-10	Male: 7.59 (1.32-19.83)	Bergh et al^12^ report risk ratio of 7.69 (95% CI, 1.67-33.3)
		Female: 8.00 (1.73-18.73)	
BED	1.2-2.0	Male: 2.08 (1.36-3.37)	Hay et al^13^ report risk ratio of 1.59 (95% CI, 1.20-2.08) for BED and BN
		Female: 1.81 (1.10-2.86)	
BN	1.2-2.0	Male: 1.75 (1.07-2.92)	
		Female: 1.74 (1.10-3.13)	
OSFED	1.2-2.0	Male: 1.80 (1.07-3.01)	Same prior search bounds as for BED and BN used
		Female: 1.83 (1.05-3.01)	
Annual eating disorder crossovers, %^a^			
AN to BED	Male: 0-1.5	Male: 1.2 (0.1-3.0)	Relative probabilities informed by PEDSnet analysis by Rodriguez et al^14^
	Female: 0-1.5	Female: 1.2 (0.1-3.1)	
AN to BN	Male: 0-0.03	Male: 0.0 (0.0-0.1)	
	Female: 0-0.4	Female: 0.3 (0.0-0.9)	
AN to OSFED	Male: 0-4.5	Male: 4.0 (0.2-10.3)	
	Female: 0-4.5	Female: 3.8 (0.4-10.4)	
BED to AN	Male: 0-1.3	Male: 1.1 (0.2-2.9)	
	Female: 0-1.2	Female: 0.5 (0.0-1.6)	
BED to BN	Male: 0-0.2	Male: 0.2 (0.0-0.4)	
	Female: 0-0.4	Female: 0.3 (0.0-0.9)	
BED to OSFED	Male: 0-4.9	Male: 5.4 (0.2-12.1)	
	Female: 0-4.7	Female: 3.4 (0.2-8.2)	
BN to AN	Male: 0-0.9	Male: 0.7 (0.1-1.9)	Assumed that BED estimates accounted for 25% of OSFED prevalence on the basis of lifetime prevalence estimates from Hudson et al^4^ and Stice et al^7^
	Female: 0-2.1	Female: 1.3 (0.1-3.4)	
BN to BED	Male: 0-1.7	Male: 1.4 (0.1-3.7)	
	Female: 0-1.7	Female: 1.4 (0.1-3.1)	
BN to OSFED	Male: 0-5.2	Male: 4.5 (0.5-9.9)	
	Female: 0-5.0	Female: 4.5 (0.4-10.1)	
OSFED to AN	Male: 0-4.0	Male: 0.4 (0.0-1.6)	
	Female: 0-3.6	Female: 0.1 (0.0-0.6)	
OSFED to BED	Male: 0-1.6	Male: 0.6% (0.1-1.6)	
	Female: 0-1.6	Female: 0.7 (0.0-2.2)	
OSFED to BD	Male: 0-0.6	Male: 0.1 (0.0-0.4)	
	Female: 0-1.3	Female: 0.4 (0.0-1.2)	
Mortality^a^			
Background mortality	Age- and sex-specific		US life tables (Arias et al^15^)
AN, SMR	4.17-8.26	Male: 6.71 (1.43-13.12)	Arcelus et al^3^ report SMR of 5.86 (95% CI, 4.17-8.26)
		Female: 6.31 (1.33-12.44)	
BED, SMR	1.46-2.52	Male: 2.01 (1.08-3.53)	Ackard et al^16^ report suicide ideation or attempt odds ratios; Arcelus et al^3^ report SMR of 1.92 (95% CI, 1.46-2.52) for EDNOS
		Female: 2.15 (1.10-3.68)	
BN, SMR	1.44-2.59	Male: 2.26 (1.19-3.49)	Arcelus et al^3^ report SMR of 1.93 (95% CI, 1.44-2.59)
		Female: 2.31 (1.09-3.64)	
OSFED, SMR	1.46-2.52	Male: 2.17 (1.15-3.81)	Arcelus et al^3^ report SMR of 1.92 (95% CI, 1.46-2.52) for EDNOS
		Female: 2.07 (1.12-3.67)	

Abbreviations: AN, anorexia nervosa; BED, binge eating disorder; BN, bulimia nervosa; EDNOS, eating disorder not otherwise specified; NA, not applicable; OSFED, other specified feeding and eating disorder; SMR, standardized mortality ratio; UI, uncertainty interval.

^a^ See eAppendix 2 in the Supplement for more details on how the parameters were calculated.

^b^ See eFigure 1 in the Supplement for more details on how the parameters were calculated.

#### Incidence and Relapse

Studies^1,4^ comparing lifetime prevalence with annual prevalence have found that the risk of ED is concentrated among subsets of people, or that they frequently persist (or recur) among the same individuals over time. Thus, heterogeneity in the risk of ED incidence or relapse is an important consideration. Individual-level risk factors for ED include genetics^17,18^ and psychiatric comorbidities, such as depression, anxiety, and substance abuse.^18^ To model the incidence of ED accounting for heterogeneity, we therefore differentiated between naive (first-time) incidence of a disorder and the probability of recurring disorders (relapse) among those with a history of ED.

For naive incidence, we set prior search bounds for AN and BN according to age-specific estimates from the 2017 Global Burden of Disease Study.^6^ For BED and OSFED naive incidence, we used estimates from a prospective community study^7^ and imputed age curves on the basis of the shape from the Global Burden of Disease Study estimates for AN and BN (eAppendix 2 in the Supplement).

For individuals with a history of ED, we modeled a risk of relapse instead. Studies^7,8,9,10,11^ have found that ED relapse is common, with rates of approximately 30% reported for each ED diagnosis in the model (eAppendix 2 in the Supplement). Although these studies provide estimates of ED relapse in the follow-up period after remission, they do not follow individuals for longer periods. To account for the fact that the risk of relapse likely decreases over time,^11^ we modeled relapse using a declining exponential function. This allowed us to model more realistic risks of relapse that are highest during the first year and then decrease over time at a rate controlled by a parameter fitted via calibration (eAppendix 2 in the Supplement).

#### Remission and Treatment

Baseline remission rates are used to model the probability of transitions back to healthy for the natural history of ED (ie, in the absence of treatment). For individuals who receive treatment for ED, we modeled a treatment effect that increases their probability of transitioning to healthy (see details later in this section). Although estimates of overall remission rates are available from a prospective study of adolescent girls,^7^ these estimates are based on individuals who have received a diagnosis of an ED and have been enrolled in a study. In the absence of diagnosis and treatment, remission rates would be expected to be lower. As a proxy for the natural history of remission, we based our prior estimates of baseline remission rates on data available from the control groups in clinical trials^12,13^ (eAppendix 2 in the Supplement).

Because the population-level estimates of ED prevalence that we used to calibrate the model include some proportion of people who receive treatment, we included parameters to model the proportion of patients with ED who receive treatment, as well as treatment efficacy for ED remission. To model the proportion of individuals who receive treatment, we based our prior search bounds on a nationally representative household survey.^4^ We modeled the effect of treatment using a rate ratio that increases the remission rate. To set prior bounds on our estimates of treatment efficacy, we used data from Cochrane systematic reviews.^13,19^ We did not find any estimates of treatment efficacy on the risk of future relapse or mortality, so we assumed that ED treatment is associated with only remission probabilities in the model (eAppendix 2 in the Supplement).

#### ED Crossovers

Transition probabilities to other EDs (ie, crossovers) were based on an analysis of data from PEDSnet,^20^ a clinical data research network of 8 children’s hospitals. On the basis of a data set of electronic medical records of approximately 10 000 patients, annual transition probabilities were estimated using survival analysis.^14^ However, because these data are conditional on patients still having ED and still seeking treatment at a children’s hospital, they likely overestimate the transition probabilities, because remission is not observed in the data set. We, therefore, rescaled these probabilities for calibration and used the relative propensity of transitioning among ED types to inform the dynamics of ED crossover in the model (eAppendix 2 in the Supplement).

#### Mortality

Background mortality (ie, mortality for healthy individuals) was modeled according to age- and sex-specific US life tables.^15^ Eating disorder–associated mortality was modeled using a standardized mortality ratio, which we multiplied by the background mortality rates to account for excess mortality for each ED state. Estimates of standardized mortality ratios used to inform prior search bounds were based on a meta-analysis of 36 studies.^3^ We assumed that the standardized mortality ratio estimated for EDNOS was applicable for the new *DSM-5* OSFED diagnosis. Although BED mortality estimates were not available, higher rates of suicidal ideation and suicide attempts have been reported among patients with BED compared with individuals without BED.^16^ We therefore used the same prior search bounds for BED and OSFED mortality (eAppendix 2 in the Supplement).

### Model Calibration

Calibration involves comparing our model simulated estimates with empirical data to find parameter sets that achieve a good fit.^21^ We calibrated the model to empirical data on the prevalence of ED at different ages on the basis of nationally representative surveys from 2007 and 2011. We fit the model to 12-month prevalence estimates of AN, BN, and BED^1,4^; cumulative lifetime prevalence estimates at age 20 years for OSFED^7^; and cumulative lifetime prevalence estimates at ages 30 to 44 years for AN, BED, and BN^4^ (eAppendix 3 in the Supplement).

A directed search algorithm (simulated annealing) was used to find good-fitting parameter sets. We scored the goodness-of-fit of each parameter set as the sum of the distance squared (ie, quadratic loss function) between each prevalence target and model simulated estimate. We weighted each prevalence target by the inverse of its standard error, allowing more precise targets to have more influence during the calibration. We ran 10 000 independent searches of 1000 iterations each, selecting the final best-fitting 100 sets (eFigure 2 in the Supplement). During calibration, if a sampled set of parameters was invalid (ie, probabilities sum to >1.0), the lower bounds of the relevant search bounds were iteratively lowered until a valid parameter set was sampled.

### Statistical Analysis

We ran 1000 simulations of 100 000 individuals to explore ED dynamics, in each simulation sampling a parameter set from the best 100 sets identified in calibration. This approach takes into account both individual-level, stochastic (first-order), and parameter (second-order) uncertainty around our estimates.^22^ We estimated age-specific annual prevalence and lifetime prevalence of each ED type from birth to age 40 years and calculated the probability of initial (naive) ED incidence by age. We also calculated the distribution of number of ED episodes per person. We report the mean and 95% uncertainty intervals (UIs), calculated as the 2.5th and 97.5th percentiles of our simulation results.

To estimate ED-associated excess mortality, we ran a counterfactual scenario in which the incidence of ED was set to 0. We also ran counterfactual scenarios in which we assumed that no individuals received treatment, or all individuals received treatment, and estimated the change in mortality. Common random numbers were used for all scenarios to reduce the variance of the estimates and enable individual-level counterfactual comparisons.^23^ The model was developed in Java software (version 1.8.0, Oracle), and statistical analyses were performed in April 2019.

## Results

### Calibration Results

The calibrated model was able to fit the targets well, with 87% of the simulated estimates falling within the target confidence intervals (eFigure 3 and eFigure 4 in the Supplement). The estimated prevalence of AN and BN was also similar to estimates from the 2017 Global Burden of Disease Study,^6^ although the model-estimated prevalence of AN tended to be higher and that of BN tended to be lower than the Global Burden of Disease Study estimates, especially at older ages (eFigure 5 in the Supplement). Calibrated model parameter values are presented in eFigures 1, 6, 7, 8, 9, and 10 in the Supplement; summaries are reported in the Table.

### ED Dynamics by Age

**Figure 1.**
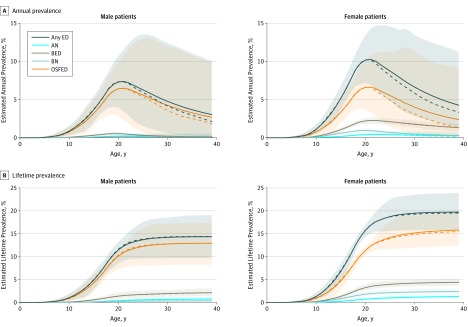
Estimated Prevalence of Eating Disorders (EDs), by Age, Sex, and Type of ED Estimated annual (A) and lifetime (B) prevalence of EDs are shown. Solid lines indicate means. Dashed lines indicate medians. Shaded areas indicate 95% uncertainty intervals. AN indicates anorexia nervosa; BED, binge eating disorder; BN, bulimia nervosa; and OSFED, other specified feeding and eating disorder.

The highest estimated mean annual prevalence of ED overall occurred at approximately age 21 years for both male (7.4%; 95% UI, 3.5%-11.5%) and female (10.3%; 95% UI, 7.0%-14.2%) individuals, with mean lifetime prevalence increasing to approximately 1 in 7 (14.3%; 95% UI, 9.7%-19.0%) for male individuals and approximately 1 in 5 (19.7%; 95% UI, 15.8%-23.9%) for female individuals by age 40 years (Figure 1). Types of ED followed a similar pattern, peaking in the early 20s and decreasing slowly in later adulthood, with OSFED comprising most ED cases (eFigure 11 in the Supplement). Although the estimated prevalence in the population continues into later ages, the initial incidence of EDs is concentrated earlier, with nearly all (95%) first-time cases occurring by age 25 years (Figure 1B).

**Figure 2.**
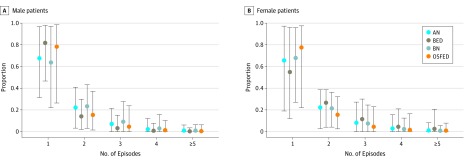
Number of Eating Disorder Episodes by Age 40 Years Among Simulated Individuals With History of Eating Disorder Numbers of eating disorder episodes for simulated male (A) and female (B) patients are shown. Circles indicate means; vertical lines, 95% uncertainty intervals. AN indicates anorexia nervosa; BED, binge eating disorder; BN, bulimia nervosa; and OSFED, other specified feeding and eating disorders.

The model estimated that most patients with EDs have 1 episode (78% of male patients [95% UI, 38%-96%]; 71% of female patients [95% UI, 38%-87%]), with very few having 5 or more recurrent episodes, and female patients more likely to have multiple episodes. Similar patterns were found by ED, which suggest that AN and BN recur more frequently among male patients, and BED is more recurrent among female patients, but the uncertainty around these estimates is large (Figure 2).

**Figure 3.**
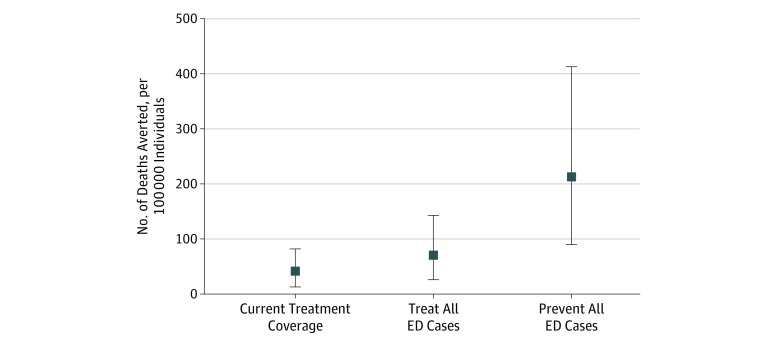
Deaths Averted by Age 40 Years per 100 000 Individuals, by Treatment Scenario Number of deaths due to eating disorders (EDs) averted by various treatment scenarios is shown. Squares indicate means; vertical lines, 95% uncertainty intervals.

Compared with no treatment, the model estimated that current treatment averts a mean of 41.7 deaths per 100 000 individuals (95% UI, 13.0-82.0 deaths per 100 000 individuals) by age 40 years (Figure 3). Increasing treatment to cover all ED cases would avert an estimated mean of 70.5 deaths per 100 000 individuals (95% UI, 26.0-143.0 deaths per 100 000 individuals) by age 40 years. In comparison, preventing all ED cases, thus eliminating ED-associated deaths, would avert an estimated mean of 213.0 deaths per 100 000 individuals (95% UI, 90.0-413.1 deaths per 100 000 individuals) by age 40 years, highlighting the magnitude of the ED mortality burden.

## Discussion

In light of the scarce data on EDs, we developed a simulation model to provide insights into ED dynamics, using a Bayesian calibration approach to synthesize currently available data from multiple sources into an internally consistent model. We found that, although the estimated annual prevalence of EDs is low compared with other conditions, the cumulative lifetime burden is high, with 14.3% of male and 19.7% of female individuals estimated to experience an ED by age 40 years. We found that risk is generally concentrated among individuals, indicating a high amount of heterogeneity.

Our findings support the idea that adolescence and young adulthood are critical periods for the initial development of ED, with nearly all (95%) first-time cases occurring by age 25 years in our simulated cohort that we modeled from birth to age 40 years. This finding suggests that prevention efforts may best be targeted to adolescent or younger individuals. However, given the risk of relapse and continued ED prevalence at later ages, diagnosis and treatment of EDs at older ages should also be a priority.

We found that increasing treatment coverage could help alleviate the burden of EDs and substantially reduce ED-associated deaths. Given that few patients with ED are estimated to receive treatment, this highlights the potential role of increased identification and treatment of those with EDs. Further research on cost-effective strategies to increase the proportion of patients with EDs who receive treatment could help to alleviate the ED disease burden.

### Limitations

Because of data limitations, we could not take into account other risk factors that may be associated with ED dynamics, such as other mental disorders, substance abuse, family history, or sexual orientation. Further research on how these factors may be associated with disease dynamics, such as incidence, remission, or mortality, would help to better contextualize the role of heterogeneity in EDs. In particular, increasing treatment coverage for high-risk individuals could potentially result in larger mortality reductions.

Second, the transportability of standardized mortality ratio and ED crossover estimates may affect our results, because the simulated populations for which they were estimated may not be comparable to older cohorts or individuals with undiagnosed EDs. We also assumed that treatment is associated with remission probabilities only, with no association with mortality or future risks of ED relapse. Although there is evidence that pharmacotherapy may be effective in preventing BED relapse,^10^ we had no data on how many patients receive such therapy or on the effectiveness of continuing therapy for other ED types. Our estimates of the association of treatment with mortality may, thus, be conservative.

Third, in using a cohort, rather than an open population model, we were unable to explore potential secular trends in ED dynamics that may have occurred over time.^24^ For example, a recent analysis^25,26^ of ED prevalence based on data from the National Epidemiologic Survey on Alcohol and Related Conditions–III found estimates of AN that were similar to previous estimates, but estimates of BED and BN that were lower for reasons that are unclear, especially because the changes in criteria from *DSM-IV* to *DSM-5* would be expected to yield higher rates.

In general, these limitations highlight the lack of data on EDs. Given the recent changes in diagnosis definitions, a lot of uncertainty in our estimates is due to the lack of data on OSFED in particular, which may include various diagnoses such as atypical AN or diabulimia. We also did not include ARFID in the model because it was not established as a diagnosis until *DSM-5*. However, our model can be updated to incorporate new data as they become available.

## Conclusions

In this decision analytical model study, estimated EDs were prevalent, with nearly 1 in 7 male and 1 in 5 female individuals estimated to have had an ED by age 40 years, with nearly all first-time cases occurring by age 25 years. These findings suggest that increasing treatment coverage could substantially reduce ED-associated mortality. Further research on cost-effective prevention and treatment strategies is needed to reduce the burden of morbidity and mortality associated with these common psychiatric disorders.

## Notes

Supplement. **eAppendix 1.** Microsimulation Model **eAppendix 2.** Model Parameters: Priors **eFigure 1.** Incidence **eAppendix 3.** Calibration **eFigure 2.** Scores **eFigure 3.** 12-Month Prevalence Targets **eFigure 4.** Cumulative Lifetime Prevalence Targets **eFigure 5.** Modeled Prevalence vs GBD 2017 Estimates **eFigure 6.** Relapse **eFigure 7.** Remission **eFigure 8.** Treatment **eFigure 9.** Interdisorder Transitions **eFigure 10.** Mortality **eFigure 11.** Annual Prevalence by Age
