# Supplementary material for: Estimation of Eating Disorders Prevalence by Age and Associations With Mortality in a Simulated Nationally Representative US Cohort
Source: JAMA Netw Open. 2019 Oct 9;2(10):e1912925. doi: 10.1001/jamanetworkopen.2019.12925 (PMC6802241; doi:10.1001/jamanetworkopen.2019.12925)
Supplement: Supplement. — eAppendix 1. Microsimulation Model eAppendix 2. Model Parameters: Priors eFigure 1. Incidence eAppendix 3. Calibration eFigure 2. Scores eFigure 3. 12-Month Prevalence Targets eFigure 4. Cumulative Lifetime Prevalence Targets eFigure 5. Modeled Prevalence vs GBD 2017 Estimates eFigure 6. Relapse eFigure 7. Remission eFigure 8. Treatment eFigure 9. Interdisorder Transitions eFigure 10. Mortality eFigure 11. Annual Prevalence by Age [file jamanetwopen-2-e1912925-s001.pdf]

Supplementary Online Content

Ward ZJ, Rodriguez P, Wright DR, Austin SB, Long MW. Estimation of eating disorders prevalence by age and associations with mortality in a simulated nationally representative US cohort. *JAMA Netw Open*. 2019;2(10):e1912925. doi:10.1001/jamanetworkopen.2019.12925

eAppendix 1. Microsimulation Model

eAppendix 2. Model Parameters: Priors

eFigure 1. Incidence

eAppendix 3. Calibration

eFigure 2. Scores

eFigure 3. 12-Month Prevalence Targets

eFigure 4. Cumulative Lifetime Prevalence Targets

eFigure 5. Modeled Prevalence vs GBD 2017 Estimates

eFigure 6. Relapse

eFigure 7. Remission

eFigure 8. Treatment

eFigure 9. Interdisorder Transitions

eFigure 10. Mortality

eFigure 11. Annual Prevalence by Age

This supplementary material has been provided by the authors to give readers additional information about their work.

© 2019 Ward ZJ et al. *JAMA Network Open.*

eAppendix 1. Microsimulation Model

We developed a microsimulation model in Java to simulate the course of eating disorders from birth until age 40. The model uses an annual cycle and follows a cohort of 100,000 individuals (50,000 males and 50,000 females) as they transition between a set of health states. We assumed that eating disorders were mutually exclusive, and modeled 6 health states in total: Healthy, Anorexia Nervosa (AN), Binge Eating Disorder (BED), Bulimia Nervosa (BN), Other Specified Feeding or Eating Disorder (OSFED), and Dead.

Using a microsimulation (i.e. individual-level) model allows us to condition transition probabilities on an individual's history of eating disorder episodes and treatment. For example, we model higher remission probabilities for individuals who receive treatment for eating disorders. Also, considering an individual's history of eating disorders allows us to model probabilities of relapse that are much higher than general incidence for people without a history of eating disorders. Empirical studies have found that the lifetime prevalence of eating disorders by middle age is generally 2-3 times higher than 12-month period prevalence at earlier ages. This suggests that the risk of eating disorders is concentrated among a subset of people who may repeatedly transition between 'Healthy' and an eating disorder state. Rather than assuming all individuals have independent, identical probabilities of eating disorder incidence, a microsimulation allows us to model this concentration of risk and take heterogeneity into account.

We calibrated the model parameters (i.e. annual probabilities of transitioning between each state) so that the predicted prevalence of eating disorders from the model (12-month period prevalence and cumulative lifetime prevalence) was consistent with empirical data. Prior probability distributions for each parameter (i.e. initial search bounds) were set based on empirical data, as described below.

© 2019 Ward ZJ et al. *JAMA Network Open.*

eAppendix 2. Model Parameters: Priors

Incidence

Estimated incidence rates for AN and BN by sex and age group were available from the Global Burden of Disease (GBD) 2017. We converted these incidence rates to annual probabilities and used the 95% CIs to inform the upper bound of the initial search bounds.

For BED and OSFED, 8-year cumulative incidence estimates were reported in Stice 2013 among a sample of 496 adolescent females. BED was reported for 13 out of 496 individuals (2.6%). To estimate the cumulative incidence of OSFED we summed cases of other reported eating disorders: Feeding or Eating Disorder-Not Elsewhere Classified (n=53), Atypical anorexia nervosa (n=14), Subthreshold bulimia nervosa (n=19), Subthreshold binge eating disorder (n=17), and Purging disorder (n=17). This yielded 120 out of 496 individuals (24.2%). Note however that these categories were not mutually exclusive in the Stice analysis, so to the extent that individuals have multiple eating disorders, summing across categories may overestimate OSFED incidence. Because we had no data on OSFED incidence or prevalence for males we constrained the incidence probabilities for males to be less than females, for which we have incidence estimates and a calibration target at age 20 (see Targets below).

We estimated 95% CIs for these estimates (BED and OSFED incidence) using Beta distributions. We then converted these estimates of 8-year cumulative incidence to annual probabilities, assuming a constant rate over the period. Lastly, we imputed age-group specific estimates by scaling the average shape of the GBD age-group estimates for AN and BN so that the mean adolescent incidence probabilities coincided with the reported estimates for BED and OSFED.

We set the lower bound for each incidence prior to 0 to encourage the model to search lower probabilities of incidence since we account for much higher probabilities of relapse among individuals with a history of eating disorders (see below). We also set the initial upper bound for the prior distribution to the estimated lower bound from the GBD for AN and BN to narrow the parameter space.

The incidence for each age group was sampled independently, but we enforced the structure of the age curves (i.e. the relative shapes of the curves were preserved during calibration). Age-specific (single-year) incidence probabilities were linearly interpolated using the midpoint of the sampled age-group probabilities.

Here we plot the prior bounds:

© 2019 Ward ZJ et al. *JAMA Network Open.*

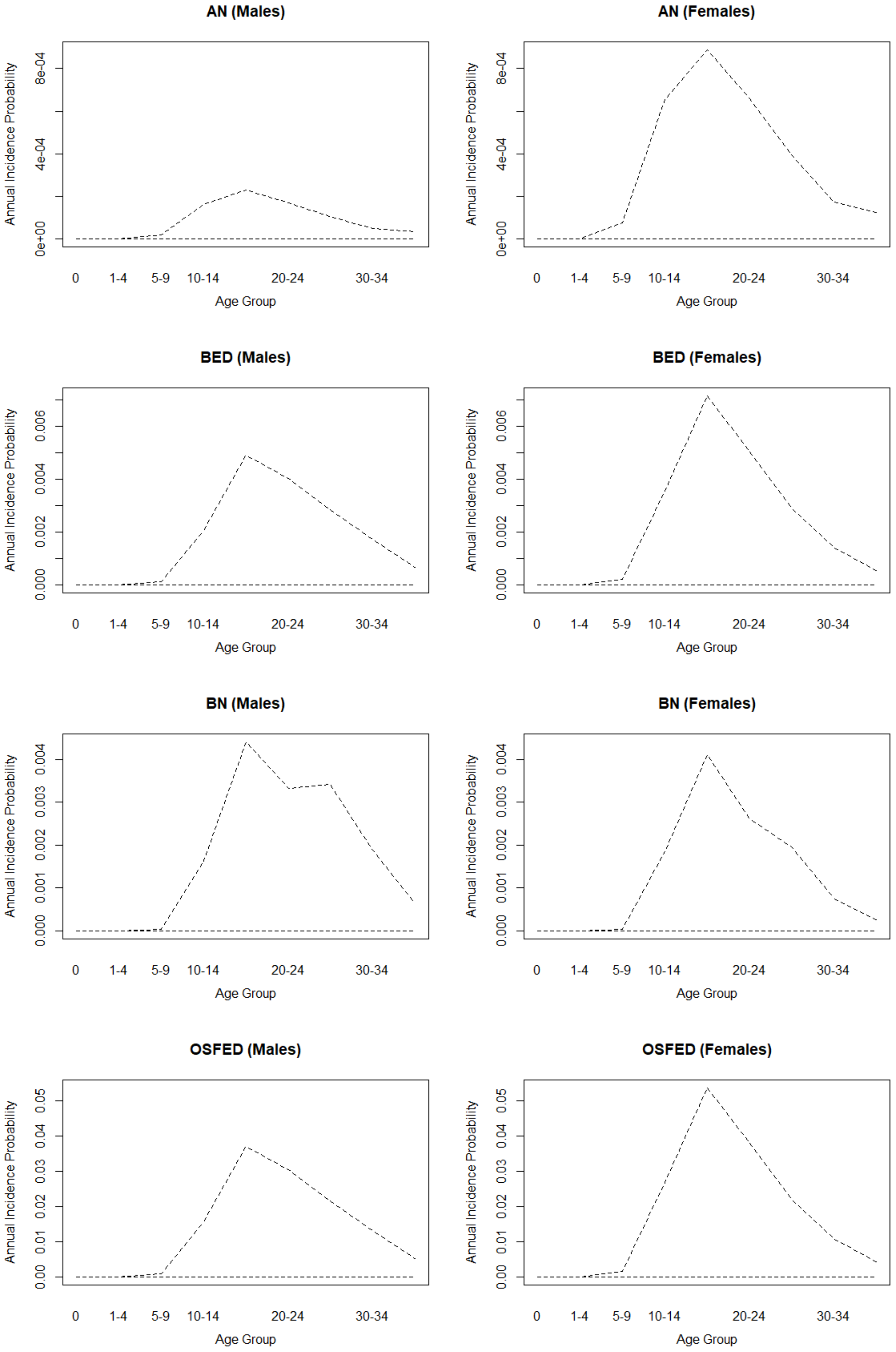

© 2019 Ward ZJ et al. *JAMA Network Open.*

References:

Global Burden of Disease Collaborative Network. Global Burden of Disease Study 2017 (GBD 2017) Results. Seattle, United States: Institute for Health Metrics and Evaluation (IHME), 2018. Available from http://ghdx.healthdata.org/gbd-results-tool.

Stice E, Marti CN, Rohde P. Prevalence, Incidence, Impairment, and Course of the Proposed DSM-5 Eating Disorder Diagnoses in an 8-Year Prospective Community Study of Young Women. J Abnorm Psychol 2013; 122(2): 445-457.

Relapse

Studies have found that the risk of eating disorders is concentrated among subsets of people. For example, a genetics study by Trace (2013) found that 50-80% of the risk for AN and BN is genetic. Another study found that about half the risk for BED is genetic (Ulfvebrand 2015).

In addition, people with eating disorders often have psychiatric co-morbidities. For example, 33-50% of AN patients have a comorbid mood disorder, such as depression, and about half of AN patients and more than half of BN patients have comorbid anxiety disorders (Ulfvebrand 2015). About half of BED patients have a comorbid mood or anxiety disorder (Ulfvebrand 2015). And about 1 in 10 BED and EDNOS (now OSFED) patients have a comorbid substance abuse disorder (Ulfvebrand 2015).

Also, a study of over 10,000 adolescents found that the ratio of 12-month to lifetime prevalence of eating disorders ranged from around 50-70% (Swanson 2011), indicating that eating disorders frequently persist (or recur) among the same individuals over time. Similar results were found in a nationally representative household survey (Hudson 2007).

This clustering of co-morbidities, together with the relatively low lifetime prevalence of eating disorders compared to 12-month prevalence, suggests that eating disorders are clustered among a subset of people. Therefore, we need to take into account higher risk of incidence (i.e. relapse) among those with a history of eating disorders.

Studies have found a substantial risk of relapse. A systematic review (Berends 2018) found that 31% of anorexia patients relapsed after treatment, with the highest risk in the first year. A relapse rate of 31% was observed during the two-year follow-up period among 48 female patients with BN who had achieved symptom control (Olmsted 1994). Another study found the rate of rapid relapse (≥ 8 episodes per month for 3 months starting within 6 months of treatment completion) was 27.6% (Olmsted 2015). A randomized trial of the efficacy of lisdexamfetamine dimesylate in adults with moderate to severe BED found that 3.7% of those on the drug met the relapse criteria, compared to 32.1% for placebo (Hudson 2017). Lastly, a prospective study (Stice 2013) found the following recurrence rates, which, though based on small samples are still informative: 25% for AN, 23% for BN, 33% for BED, and around 20%-30% for other eating disorders.

Given these findings, we set initial search bounds for the annual probability of relapse to 5%-40%. We constrained OSFED relapse probabilities for males to be lower than females since we do not have a calibration target for males with OSFED.

We assume that the probability of relapse declines over time. We modeled this using a declining exponential function $p_t = p_0 * \alpha^t$ where p_0 is the probability of relapse in the first year after remission, t is the number of years since remission, and α is a parameter in $[0,1]$ that controls how quickly the probability declines over time - smaller values of α result in faster declines. We set a uniform prior on α over $[0,1]$.

© 2019 Ward ZJ et al. *JAMA Network Open.*

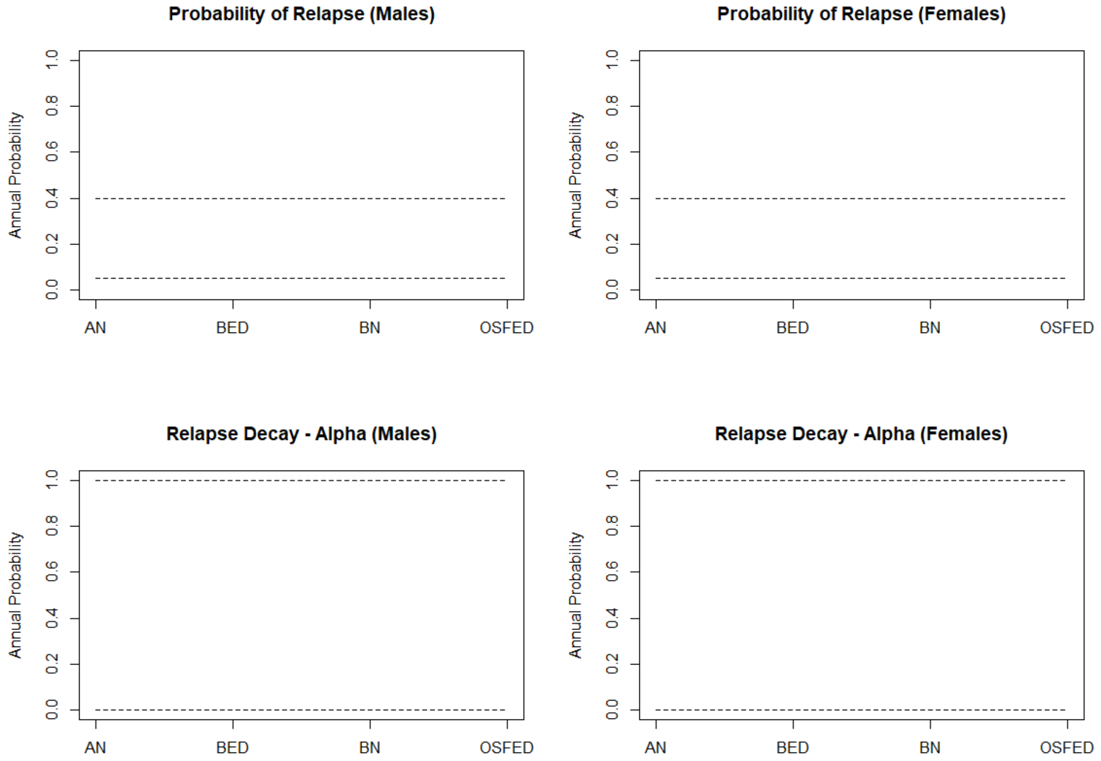

References:

Berends T, Boonstra N, van Elburg A. Relapse in anorexia nervosa: a systematic review and meta-analysis. Curr Opin Psychiatry 2018; 31(6): 445-455.

Hudson JI, Hiripi E, Pope HG, Kessler RC. The Prevalence and Correlates of Eating Disorders in the National Comorbidity Survey Replication. Biol Psychiatry 2007; 61(3): 348-358.

Hudson JI, McElroy SL, Ferreira-Cornwell MC, Radewonuk J, Gasior M. Efficacy of lisdexamfetamine in adults with moderate to severe binge-eating disorder: a randomized clinical trial. JAMA Psychiat 2017; 74: 903-910.

Olmsted MP, Kaplan AS, Rockert W. Rate and prediction of relapse in bulimia nervosa. Am J Psychiatry 1994; 151(5): 738-43.

Olmsted MP, MacDonald DE, McFarlane T, Trottier K, Colton P. Predictors of rapid relapse in bulimia nervosa. Int J Eat Disord 2015; 48(3): 337-40.

Stice E, Marti CN, Rohde P. Prevalence, Incidence, Impairment, and Course of the Proposed DSM-5 Eating Disorder Diagnoses in an 8-Year Prospective Community Study of Young Women. J Abnorm Psychol 2013; 122(2): 445-457.

Swanson SA, Crow SJ, Le Grange D, Swendsen J, Merikangas KR. Prevalence and Correlates of Eating Disorders in Adolescents. Arch Gen Psychiatry 2011; 68(7): 714-723.

© 2019 Ward ZJ et al. *JAMA Network Open.*

Trace SE, Baker JH, Peñas-Lledó E, Bulik CM. The genetics of eating disorders. Annual Review of Clinical Psychology 2013; 9: 589-620.

Ulfvebrand S, Birgegard A, Norring C, Hogdahl L, von Hausswolff-Juhlin Y. Psychiatric comorbidity in women and men with eating disorders results from a large clinical database. Psychiatry Research 2015; 230(2): 294-299.

© 2019 Ward ZJ et al. *JAMA Network Open.*

Remission

Baseline remission rates are used to model the probability of transitions back to Healthy in the 'natural history', that is, in the absence of treatment. For individuals who are treated for eating disorders we model a treatment effect that increases their probability of transitioning to Healthy (see below).

Remission estimates were available from a prospective study of 496 adolescent females (Stice 2013).
Three out of the 4 participants (75%) with AN, 13/13 (100%) participants with BN, and 14/15 (93%) participants with BED showed remission within 1 year. Based on reported data, we calculated that 117/128 (91%) individuals with other eating disorders (which we combined as a proxy for OSFED) were reported in remission after 1 year. We present the breakdown of the other eating disorders reported in Stice 2013 here:

Diagnosis	Remission	Total
FED-NEC	51	57
Atypical AN	10	14
Subthreshold BN	22	22
Subthreshold BED	18	18
PD	16	17
Total	117	128

However, these estimates are based on individuals who have been identified with ED and enrolled in a trial. Rates of remission are expected to be lower in the absence of diagnosis and treatment. Indeed, clinical trials find much lower remission rates among controls. For example, Bergh 2002 found that 10/11 (91%) treated participants with AN were in remission after a median of 14.4 months of treatment, while none of the 8 participants assigned to the delayed-treatment control group went into remission during the 21.6-months observation period. A Cochrane Review (Hay 2009) of 8 studies of psychological treatments for BN and binging found that only around 5% (10/172) of participants in the control group were in remission at the end of treatment. Based on the evidence, we thus assume that remission rates are quite low in the absence of treatment, and set priors for the annual probability of remission between 0-10%.

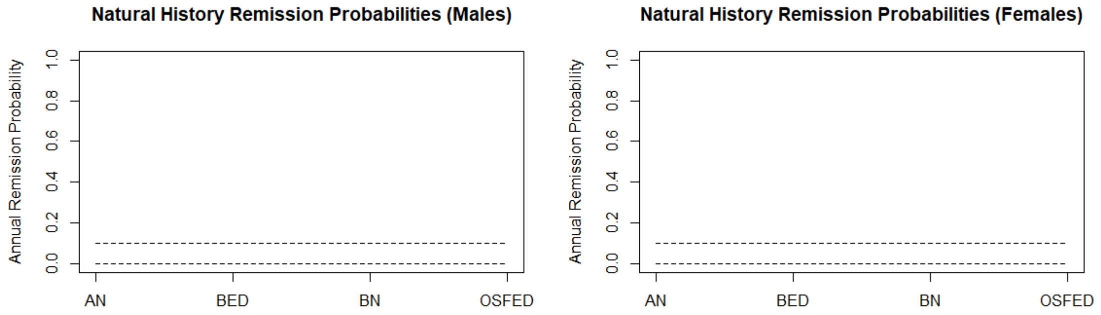

© 2019 Ward ZJ et al. *JAMA Network Open.*

References:

Bergh C, Brodin U, Lindberg G, Södersten P. Randomized controlled trial of a treatment for anorexia and bulimia nervosa. Proc Natl Acad Sci U S A. 2002; 99(14): 9486-91.

Hay PPJ, Bacaltchuk J, Stefano S, Kashyap P. Psychological treatments for bulimia nervosa and binging. Cochrane Database Syst Rev 2009; 4: CD000562.

Stice E, Marti CN, Rohde P. Prevalence, Incidence, Impairment, and Course of the Proposed DSM-5 Eating Disorder Diagnoses in an 8-Year Prospective Community Study of Young Women. J Abnorm Psychol 2013; 122(2): 445-457.

© 2019 Ward ZJ et al. *JAMA Network Open.*

Treatment

Estimates of eating disorder prevalence are based on data from the population, in which some proportion of people have received or are currently receiving treatment for eating disorders, which would change their probabilities of remission (see above). In the model we therefore assume that some proportion of people with eating disorders are on treatment, and that this increases their probabilities of remission. We model the proportion on treatment informed by data from a nationally representative household survey (Hudson 2007) and implement treatment efficacy as a remission rate ratio that is inferred via calibration.

Proportion on Treatment
The 12-month probability of treatment for females is reported in Hudson 2007 (Table 8b) for BN and BED, while only the lifetime probability of treatment is available for AN. We constructed search bounds for BN and BED based on the reported estimates and standard errors, and used the ratios of lifetime treatment for BN and BED to 12-month treatment to estimate 12-month treatment for AN. We see that the 12-month treatment proportion is about half of the lifetime probability for BN and BED, so we assume that it is similar for AN. We also assume that the search bounds for OSFED are the same as for AN in the absence of any data. We summarize the initial search bounds below:

Disorder	Females - Estimate	LB	UB	Notes
AN	0.15	0	0.3	Assume 50% of lifetime
BED	0.316	0.1	0.53	Based on 95% CI
BN	0.171	0	0.37	Based on 95% CI
OSFED	0.15	0	0.3	Assume same as AN

Similar estimates are available for males (Hudson 2007, Table 8c), but 12-month treatment is only reported for BED. We therefore estimated 12-month treatment probabilities for males based on lifetime probabilities as described above:

Disorder	Males - Estimate	LB	UB	Notes
AN	0.25	0	0.5	Assume 50% of lifetime
BED	0.215	0	0.51	Based on 95% CI
BN	0.15	0	0.3	Assume 50% of lifetime
OSFED	0.15	0	0.3	Assume same as Female OSFED

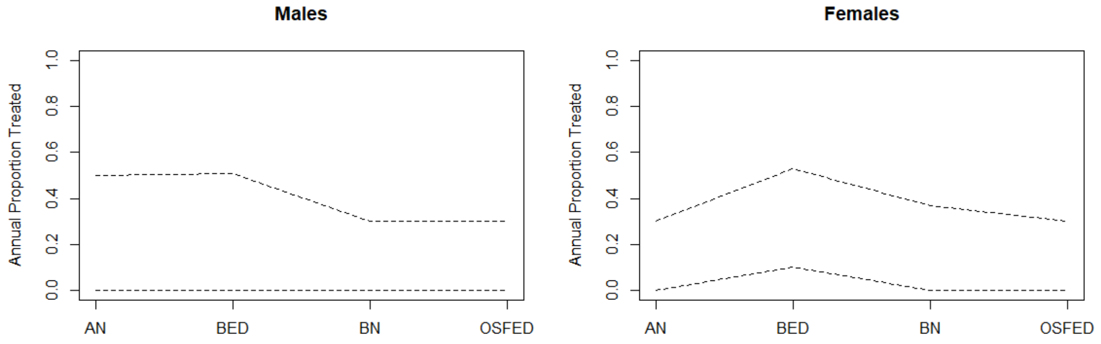

Treatment Efficacy

Estimates of treatment efficacy were based on two Cochrane systematic reviews.

Hay 2009 compared cognitive behavioral therapy (CBT) for bulimia nervosa and binging versus no treatment/waiting list. Eight studies based on 349 participants yielded a relative risk of no remission (i.e. not 100% binge free) of 0.69 (95% CI 0.61 to 0.79) in favor of CBT. We used the inverse to estimate the relative risk of remission: 1.45 (95% CI 1.26 to 1.64). A comparison of any psychotherapy (other than CBT) versus no treatment/waiting list based on six studies with 291 participants found a relative risk of no remission of 0.63 (95% CI 0.48 to 0.83). Taking the inverse yields remission RRs of 1.59 (95% CI 1.20 to 2.08). Based on these estimates we set priors of 1.2 to 2.0 for treatment efficacy for BN and BED.

Hay 2015 examined outpatient individual psychological therapy for adults with AN. However, estimates for treatment efficacy compared to no treatment were not available except for one small study (Bergh 2002) in which 10 of 11 treated participants were in remission after a median of 14.4 months of treatment, while none of the 8 participants assigned to the delayed-treatment control group went into remission during the 21.6-months observation period, yielding a RR of 7.69 (95% CI 1.67 to 33.3). Similar time to remission was observed in the study for AN, BN, and EDNOS. We therefore used the same priors for OSFED as for BN and BED, and set wide priors from 1.5 to 10 for AN informed by the magnitude in the Bergh study.

We did not find any estimates of treatment efficacy on risk of future relapse or mortality, so we assumed that treatment only affects remission probabilities in the model.

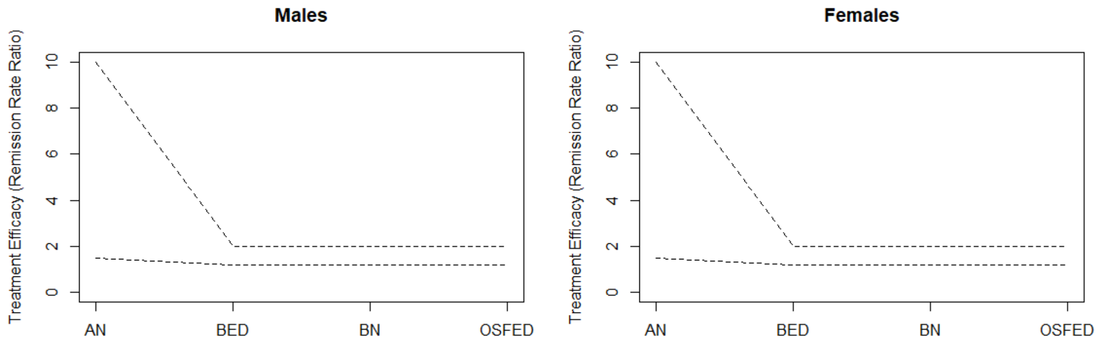

© 2019 Ward ZJ et al. *JAMA Network Open.*

References:

Bergh C, Brodin U, Lindberg G, Södersten P. Randomized controlled trial of a treatment for anorexia and bulimia nervosa. Proc Natl Acad Sci U S A. 2002; 99(14): 9486-91.

Hay PPJ, Bacaltchuk J, Stefano S, Kashyap P. Psychological treatments for bulimia nervosa and binging. Cochrane Database Syst Rev 2009; 4: CD000562.

Hay PJ, Claudino AM, Touyz S, Abd Elbaky G. Individual psychological therapy in the outpatient treatment of adults with anorexia nervosa. Cochrane Database Syst Rev 2015; 7: CD003909.

Hudson JI, Hiripi E, Pope HG, Kessler RC. The Prevalence and Correlates of Eating Disorders in the National Comorbidity Survey Replication. Biol Psychiatry 2007; 61(3): 348-358.

© 2019 Ward ZJ et al. *JAMA Network Open.*

Interdisorder Transitions

Data were obtained from PEDSnet, a clinical data research network. PEDSnet represents a consortium that includes eight children's hospitals (Boston Children's Hospital, Children's Hospital of Philadelphia, Cincinnati Children's Hospital Medical Center, Children's Hospital Colorado, Nationwide Children's Hospital, Nemours Children's Health System, Seattle Children's Hospital, and St. Louis Children's Hospital). Condition concept IDs included in patient visit conditions and problem lists were mapped to the eating disorder health states used in the model. Probabilities of transitioning between health states in the year after diagnosis were estimated using survival analysis.

As detailed in Rodriguez et al., transition probabilities in the first year were estimated based on 84,909 observations on 9,713 individuals. There were 76,311 observations on 8,568 females and 8,598 observations on 1,145 males. While the data contains a few visits as early as 2002 and as late as 2018, over 90% of the visits occurred between 2009 and 2017.

Note that the estimated transition probabilities from this dataset are conditional on still having an ED and still seeking treatment at a participating children's hospital. Therefore, all of these probabilities need to be scaled down. However, they are still useful to inform the relative disease dynamics. For example, the data reveal that AN to BN transitions are rare, a finding supported by the different behaviors associated with these diagnoses. Because this sample is comprised entirely of individuals receiving treatment we expect remission rates to be quite high. For example, Stice 2013 reports one-year remission rates of 75-100%. We therefore scaled these probabilities down by 90% when constructing prior bounds.

Estimates for BED were not available, so we assumed that OSFED included BED. Based on lifetime prevalence estimates from Stice 2013 and Hudson 2007, we assume that BED accounts for about 1/4 of BED+OSFED and split the probabilities accordingly.

References:
Rodriguez P, Ward Z, Long M, Austin SB, Wright D. Using Multi-State Modeling to Estimate Transition Probabilities for Microsimulation Models. Poster presented at: International Health Economics Association (iHEA) World Congress; July 13-17, 2019; Basel, Switzerland.

© 2019 Ward ZJ et al. *JAMA Network Open.*

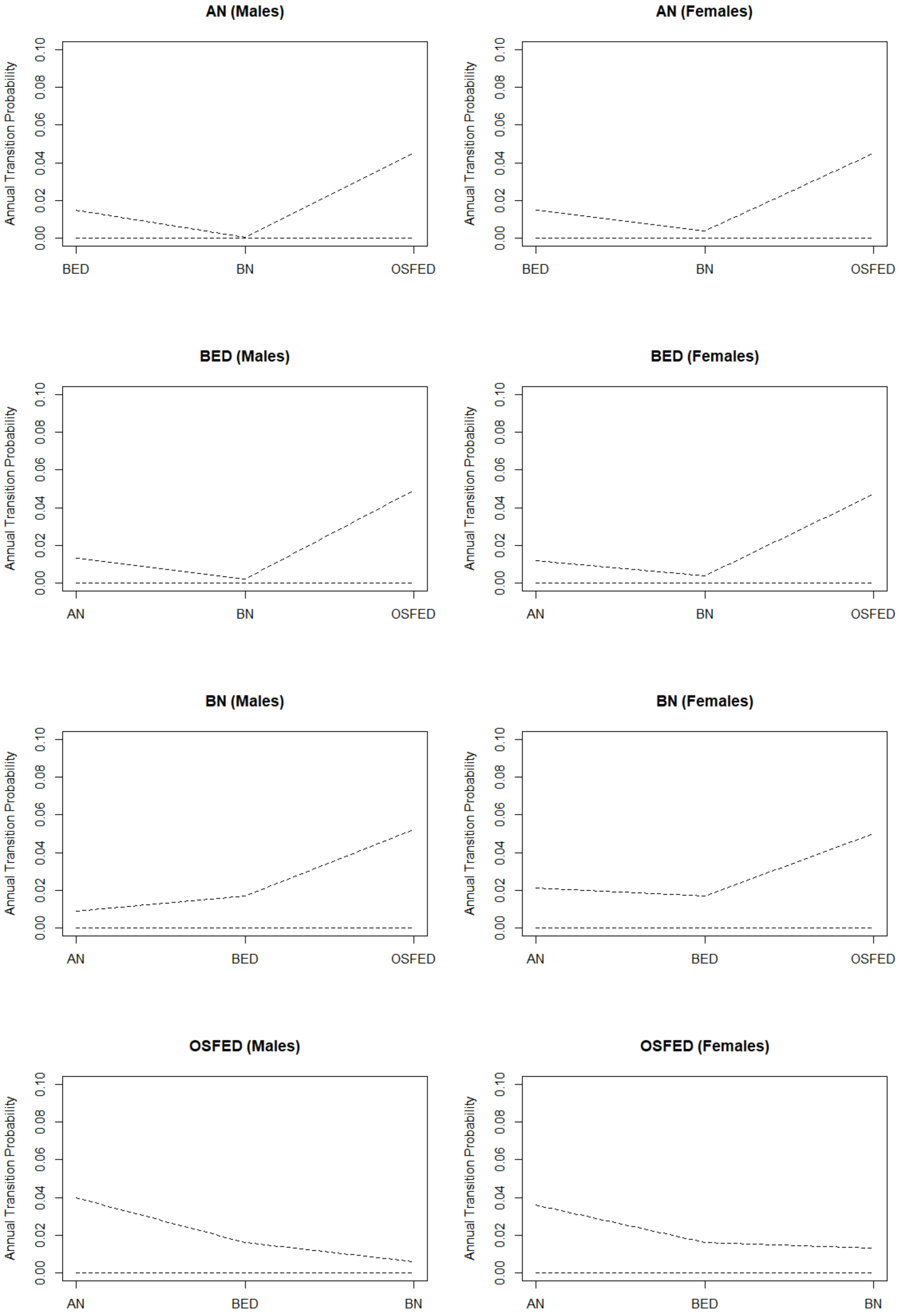

© 2019 Ward ZJ et al. *JAMA Network Open.*

Mortality

Background mortality (i.e. mortality for Healthy) was modeled based on sex-/age-specific US lifetables for 2012 (Arias 2016).

Mortality estimates were based on a meta-analysis of 36 studies (Arcelus 2011) which reported the following standardized mortality ratios (SMR):

Diagnosis	SMR
AN	5.86 (95% CI 4.17-8.26)
BN	1.93 (95% CI 1.44-2.59)
EDNOS	1.92 (95% CI 1.46-2.52)

We assumed that the SMR estimated for EDNOS was applicable for OSFED in our model.

A systematic literature review for BED (Ágh 2015) found that although BED mortality was not reported in any of the included studies, one study found that the ORs for suicidal ideation and attempt were 2.6 and 3.1, respectively, comparing BED to asymptomatic individuals (Ackard 2011). We therefore used the same EDNOS SMR bounds from Arcelus 2011 for BED.

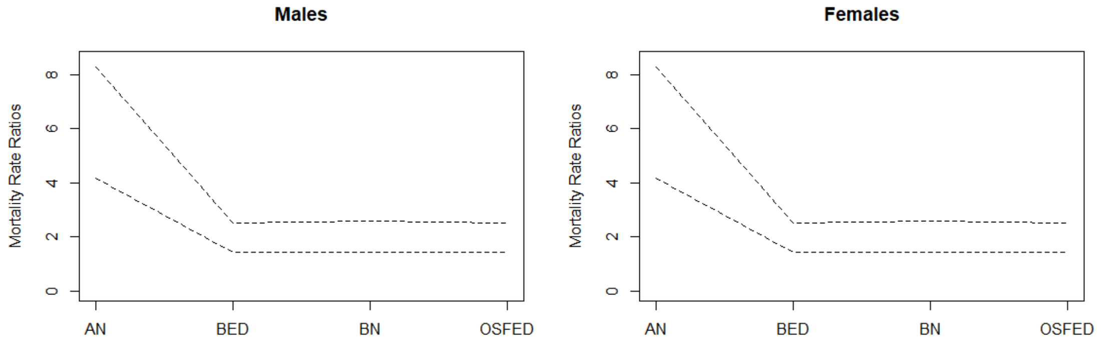

References:

Ágh T, Kovács G, Pawaskar M, Supina D, Inotai A, Vokó Z. Epidemiology, health-related quality of life and economic burden of binge eating disorder: a systematic literature review. Eat Weight Disord 2015; 20: 1-12.

Ackard DM, Fulkerson JA, Neumark-Sztainer D. Psychological and behavioral risk profiles as they relate to eating disorder diagnoses and symptomatology among a school-based sample of youth. Int J Eat Disord 2011; 44: 440-446.

Arcelus J, Mitchell AJ, Wales J, Nielsen S. Mortality Rates in Patients With Anorexia Nervosa and Other Eating Disorders: A Meta-analysis of 36 Studies. Arch Gen Psychiatry 2011; 68(7): 724-731.

Arias E, Heron M, Xu J. United States Life Tables, 2012. National Vital Statistics Reports 2016; 65(8).

© 2019 Ward ZJ et al. *JAMA Network Open.*

eFigure 1. Incidence

Black lines: Prior Search Bounds, Blue lines: Top 100 Parameter Sets

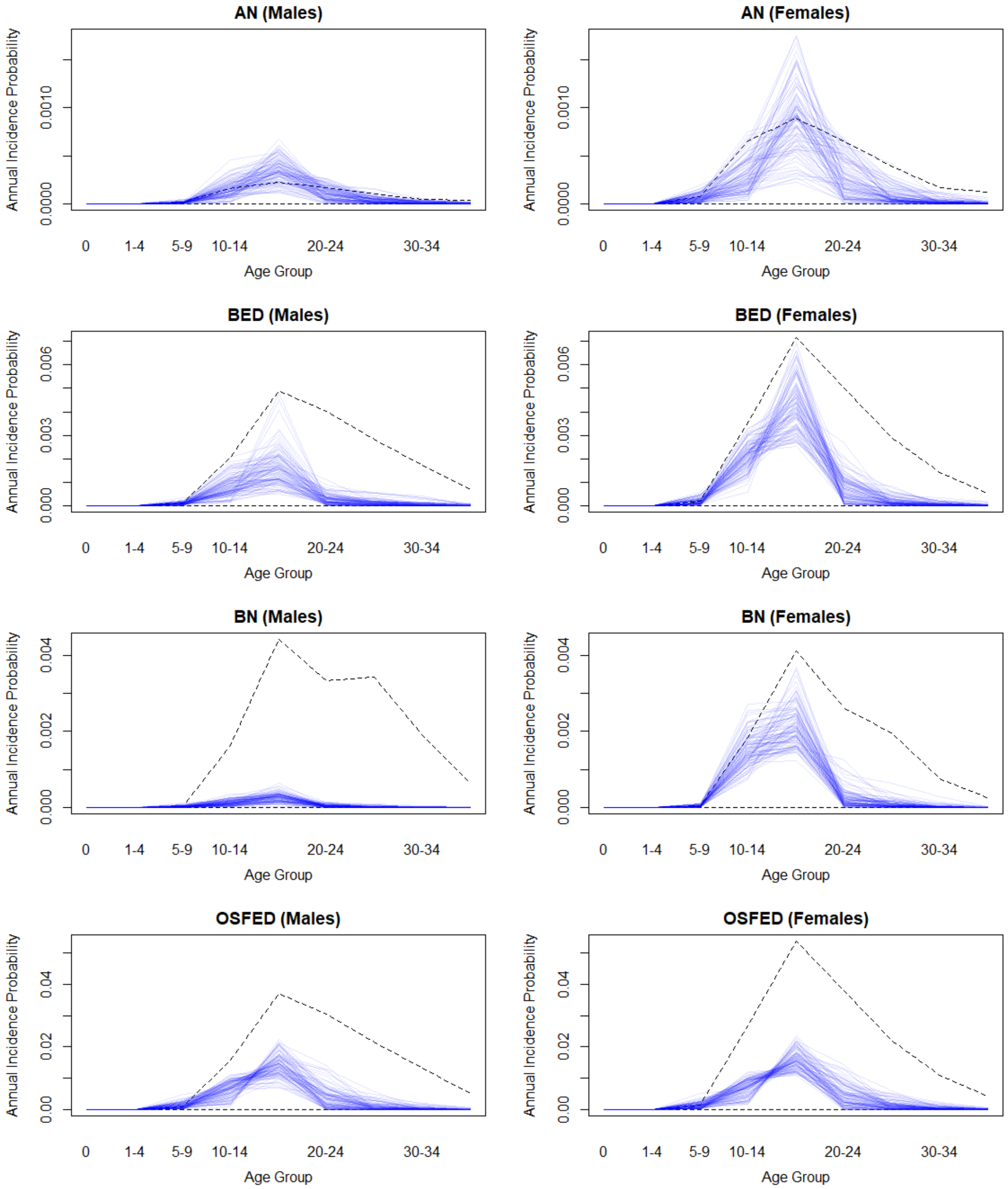

© 2019 Ward ZJ et al. *JAMA Network Open.*

eAppendix 3. Calibration

We calibrated the model to fit to empirical data on the prevalence of eating disorders by age. We fit to 12-month prevalence estimates at different ages, and cumulative lifetime prevalence estimates at age 40.

A directed search algorithm (simulated annealing) was used to find good-fitting parameter sets. We used a greedy implementation of simulated annealing where the variance of the proposal distribution followed an exponential cooling schedule ($\sigma = \alpha^t$, where t is the iteration in the search chain and we set $\alpha = 0.996$). This approach allowed the search algorithm to better explore the parameter space (i.e. larger step sizes) early in the search chain, and iteratively fine-tune the parameters (i.e. smaller step sizes) as the search chain progressed. We ran 10,000 independent searches of 1,000 iterations each, selecting the final best-fitting 100 sets.

During calibration, if a sampled set of parameters was invalid (i.e. probabilities sum to more than 1.0) the lower bounds of the relevant search bounds were iteratively lowered until a valid parameter set was sampled.

Targets

12-month Prevalence

Prevalence targets were available for different ages. Adolescent estimates were available from a nationally-representative sample of 10,123 adolescents aged 13 to 18 years (Swanson 2011). Estimates for older ages were available from a nationally-representative household survey of over 9,000 people (Hudson 2007), for the age groups 18-29 and 30-44. Because we model a cohort of individuals (i.e. everyone is the same age as the model progresses), we assumed that these estimates were representative of the midpoint of the reported age groups. That is, we compared our model predictions at single-year ages to the estimates by age group. Target ages were evaluated at the beginning of the cycle, before any transitions occurred for that age.

Model Age	Sex	AN Prev. % (SE)	BN Prev. % (SE)	BED Prev. % (SE)	Source
16	Both	0.2 (0.05)	0.6 (0.15)	0.9 (0.16)	Swanson 2011
	Male	0.2 (0.08)	0.3 (0.22)	0.4 (0.09)	Age Group: 13-18
	Female	0.1 (0.06)	0.9 (0.17)	1.4 (0.33)	
23	Both	—	0.3 (0.2)	1.4 (0.4)	Hudson 2007
	Male	—	—	0.1 (0.1)	Age Group: 18-29
	Female	—	0.6 (0.3)	2.4 (0.7)	
37	Both	—	0.4 (0.2)	1.1 (0.3)	Hudson 2007
	Male	—	—	0.9 (0.4)	Age Group: 30-44

© 2019 Ward ZJ et al. *JAMA Network Open.*

	Female	—	0.7 (0.3)	1.3 (0.4)	

Cumulative Lifetime Prevalence

Stice 2013 reports lifetime prevalence at age 20 for 496 adolescent females, finding that 57 (11.5%) had Feeding and Eating Disorders Not Elsewhere Classified (FED-NEC), which we use as a proxy for OSFED. Although OSFED also includes other diagnoses such as as atypical AN which are also reported in Stice, because the categories are not mutually exclusive we did not want to overestimate the prevalence of OSFED. Our estimates of OSFED prevalence by age 20 may thus be conservative. We constructed 95% CIs using a Beta distribution.

The following estimates of cumulative lifetime prevalence are from Hudson 2007 (Appendix Table 1) for the age group 30-44. We assumed that these estimates were representative of 40-year-olds in our model.

Sex	AN % (SE)	BED % (SE)	BN % (SE)
Males	0.6 (0.4)	2.5 (0.8)	0.1 (0.1)
Females	0.9 (0.5)	3.7 (1.0)	2.0 (0.6)
Total	0.8 (0.3)	3.1 (0.7)	1.1 (0.3)

References:

Hudson JI, Hiripi E, Pope HG, Kessler RC. The Prevalence and Correlates of Eating Disorders in the National Comorbidity Survey Replication. Biol Psychiatry 2007; 61(3): 348-358.

Swanson SA, Crow SJ, Le Grange D, Swendsen J, Merikangas KR. Prevalence and Correlates of Eating Disorders in Adolescents. Arch Gen Psychiatry 2011; 68(7): 714-723.

eFigure 2. Scores

We scored the goodness-of-fit of each parameter set as the sum of the distance-squared (i.e. quadratic loss function) between each prevalence target and model prediction. We weighted each prevalence target by the inverse of its standard error, allowing more precise targets to have more influence during the calibration.

We selected the 100 best-fitting parameter sets to use in the final model, highlighted in red below.

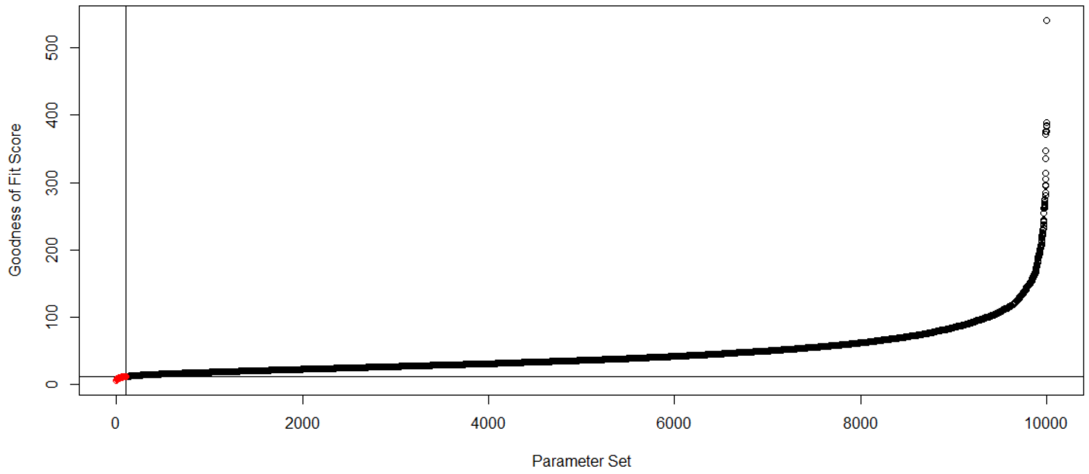

© 2019 Ward ZJ et al. *JAMA Network Open.*

eFigure 3. 12-Month Prevalence Targets

Black lines: Calibration Target 95% CIs, Blue lines: Top 100 Parameter Sets

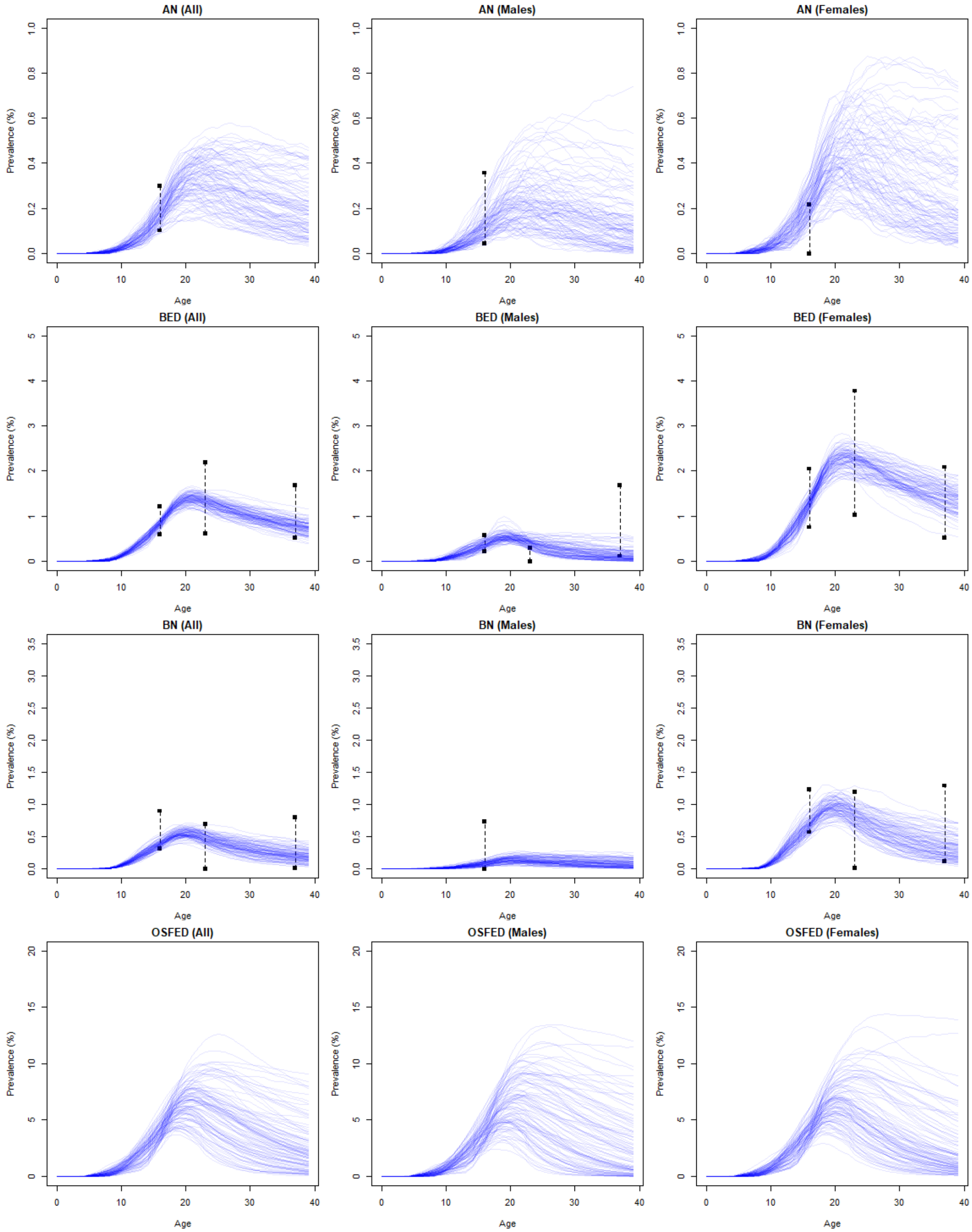

© 2019 Ward ZJ et al. *JAMA Network Open.*

eFigure 4. Cumulative Lifetime Prevalence Targets

Black lines: Calibration Target 95% CIs, Blue lines: Top 100 Parameter Sets

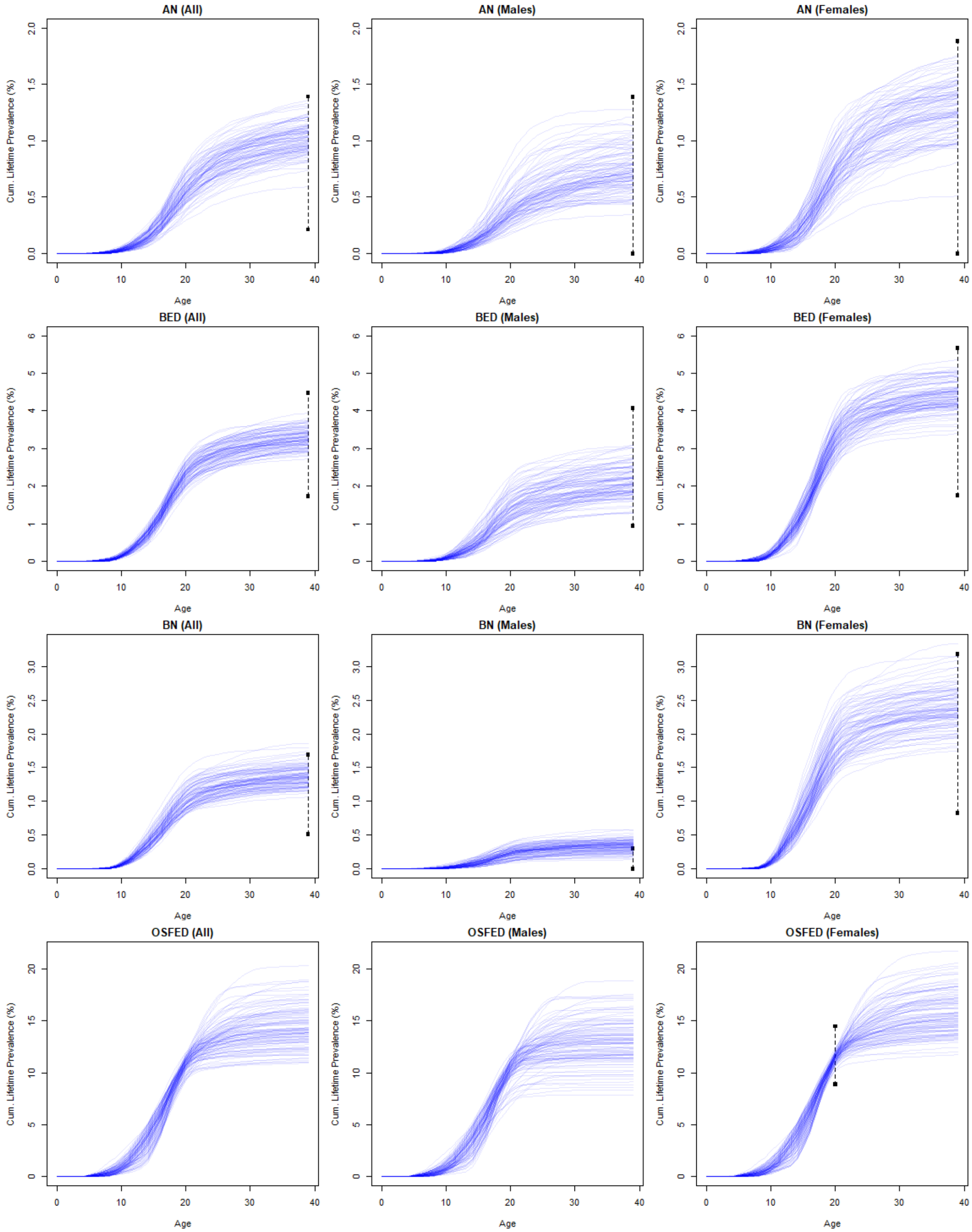

© 2019 Ward ZJ et al. *JAMA Network Open.*

eFigure 5. Modeled Prevalence vs GBD 2017 Estimates

Black lines: GBD 95% CIs, Blue lines: Top 100 Parameter Sets

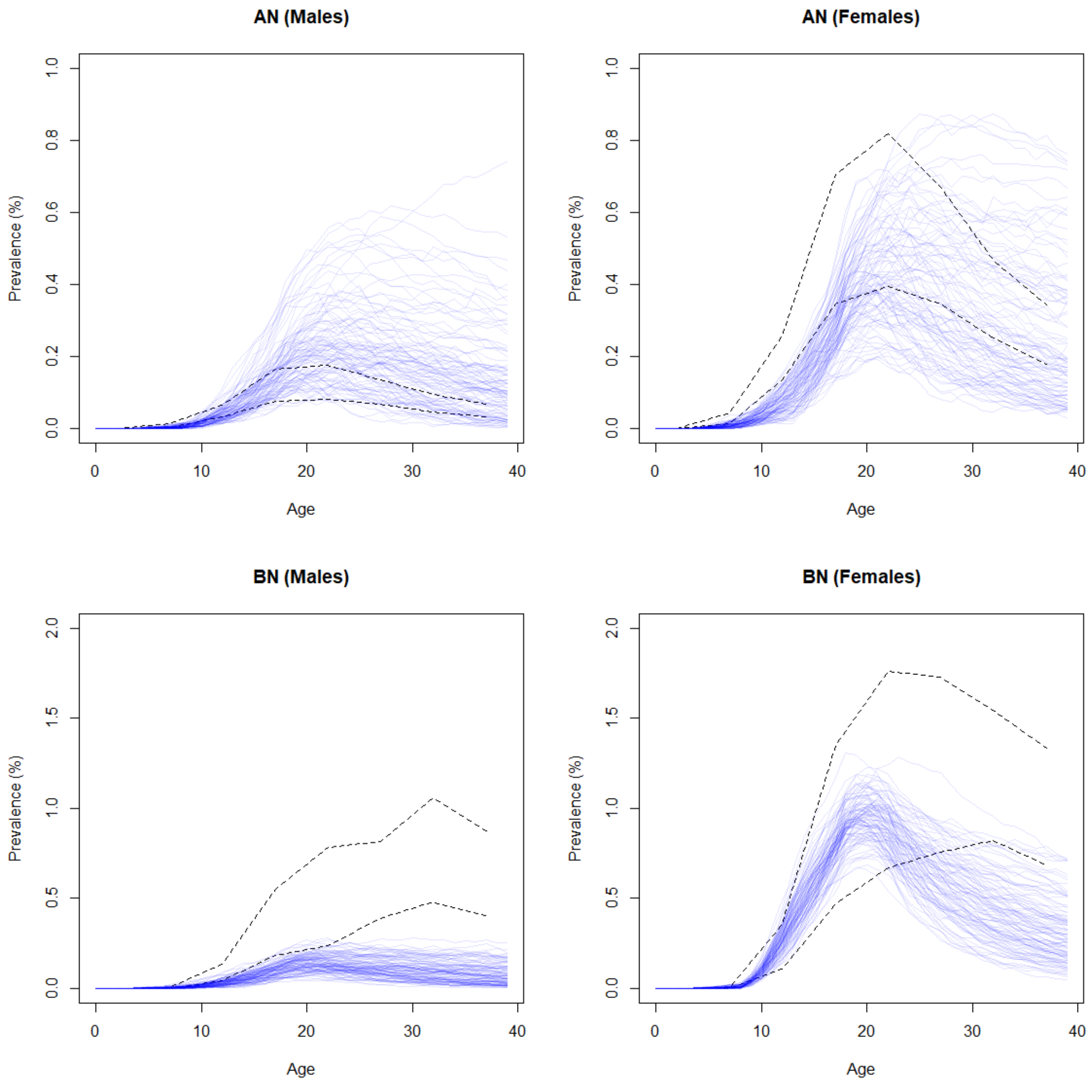

© 2019 Ward ZJ et al. *JAMA Network Open.*

eFigure 6. Relapse

Black lines: Prior Search Bounds, Blue lines: Top 100 Parameter Sets

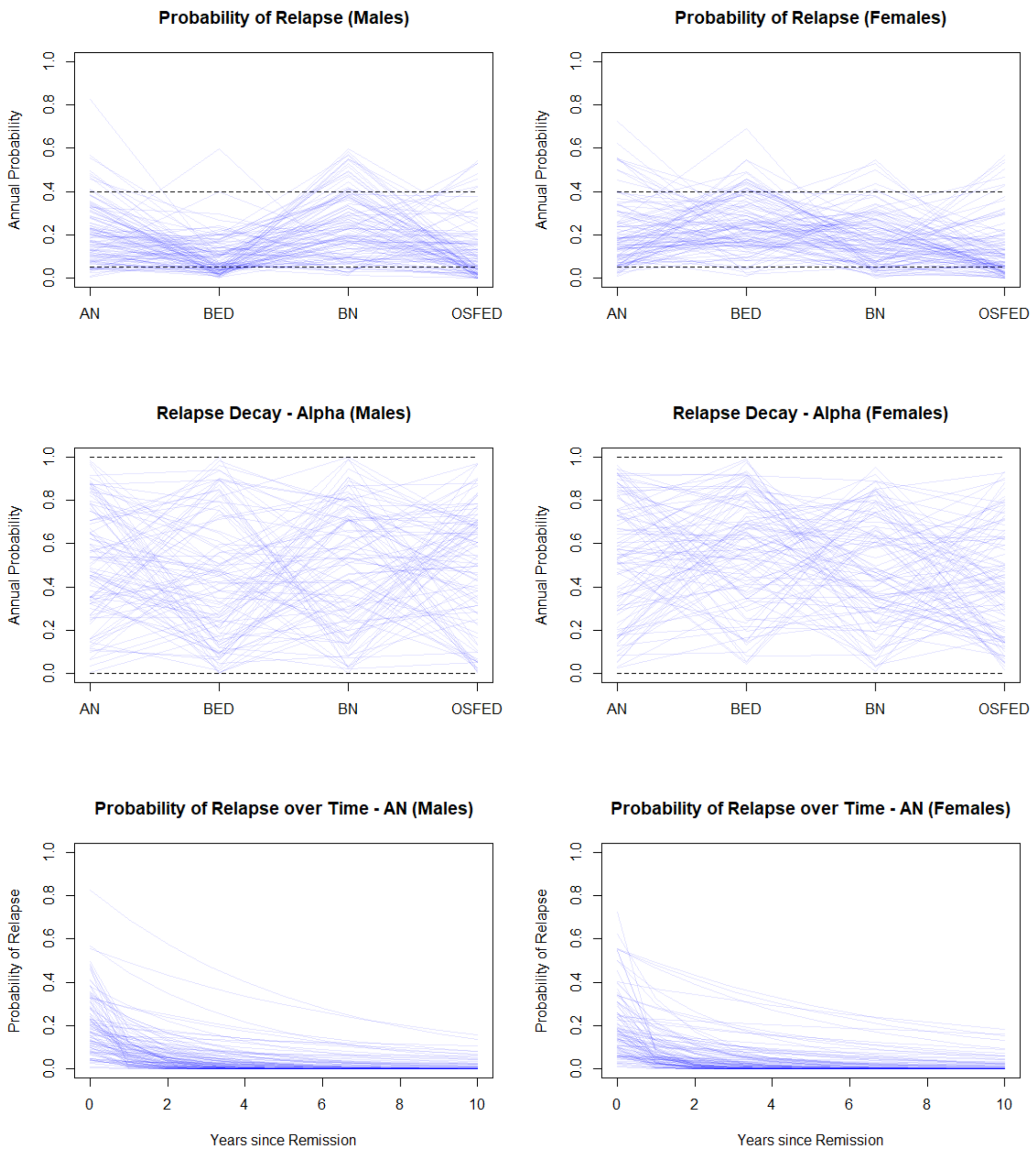

© 2019 Ward ZJ et al. *JAMA Network Open.*

Probability of Relapse over Time - BED (Males)

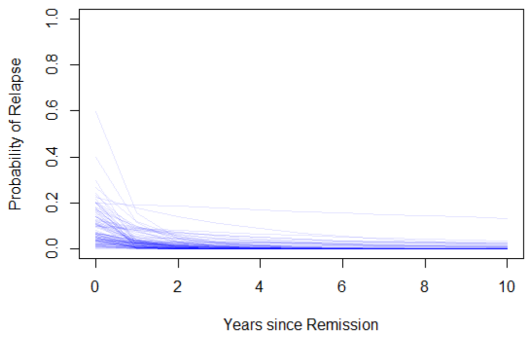

Probability of Relapse over Time - BED (Females)

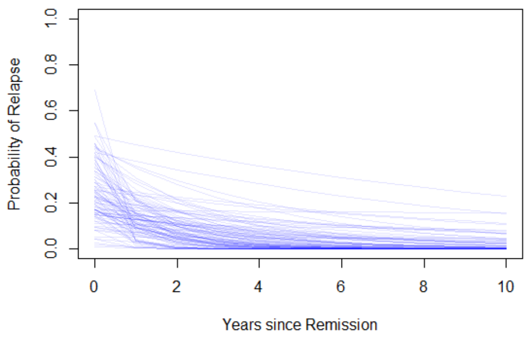

Probability of Relapse over Time - BN (Males)

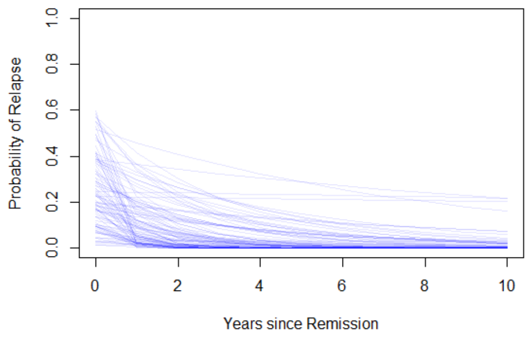

Probability of Relapse over Time - BN (Females)

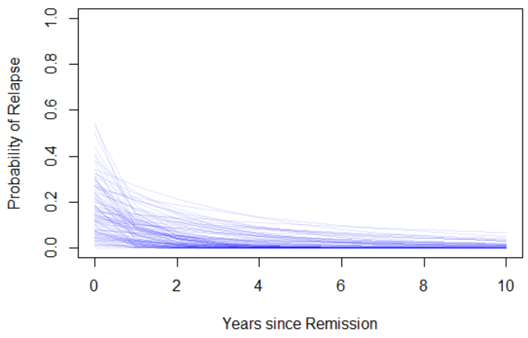

Probability of Relapse over Time - OSFED (Males)

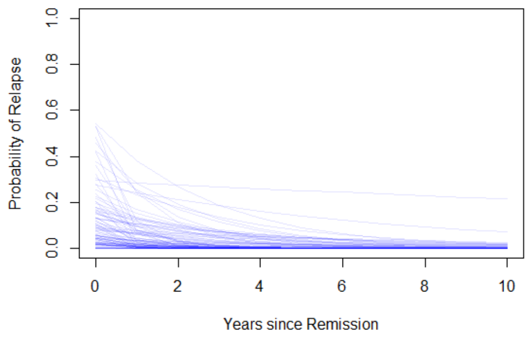

Probability of Relapse over Time - OSFED (Females)

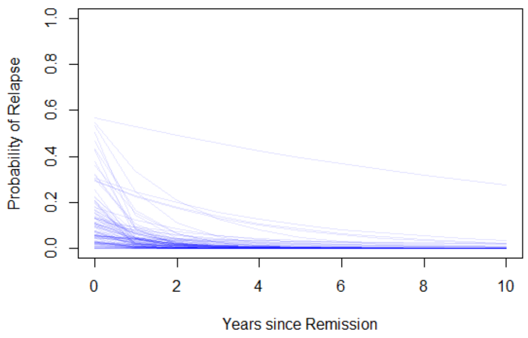

© 2019 Ward ZJ et al. *JAMA Network Open.*

eFigure 7. Remission

Black lines: Prior Search Bounds, Blue lines: Top 100 Parameter Sets

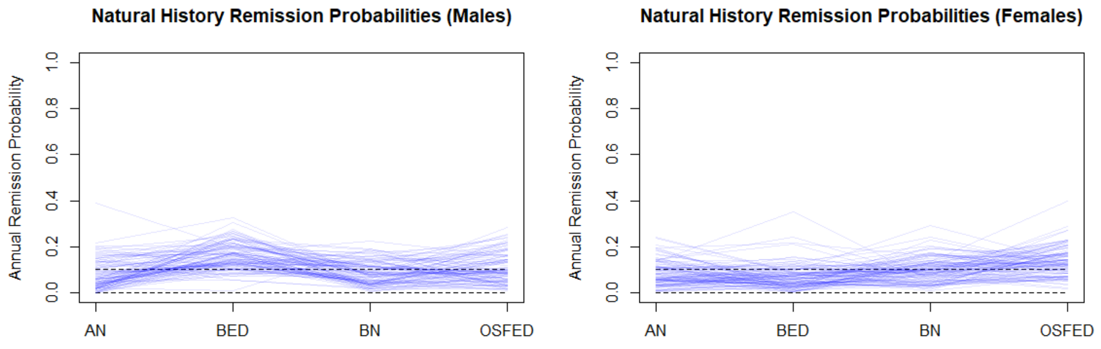

eFigure 8. Treatment

Black lines: Prior Search Bounds, Blue lines: Top 100 Parameter Sets

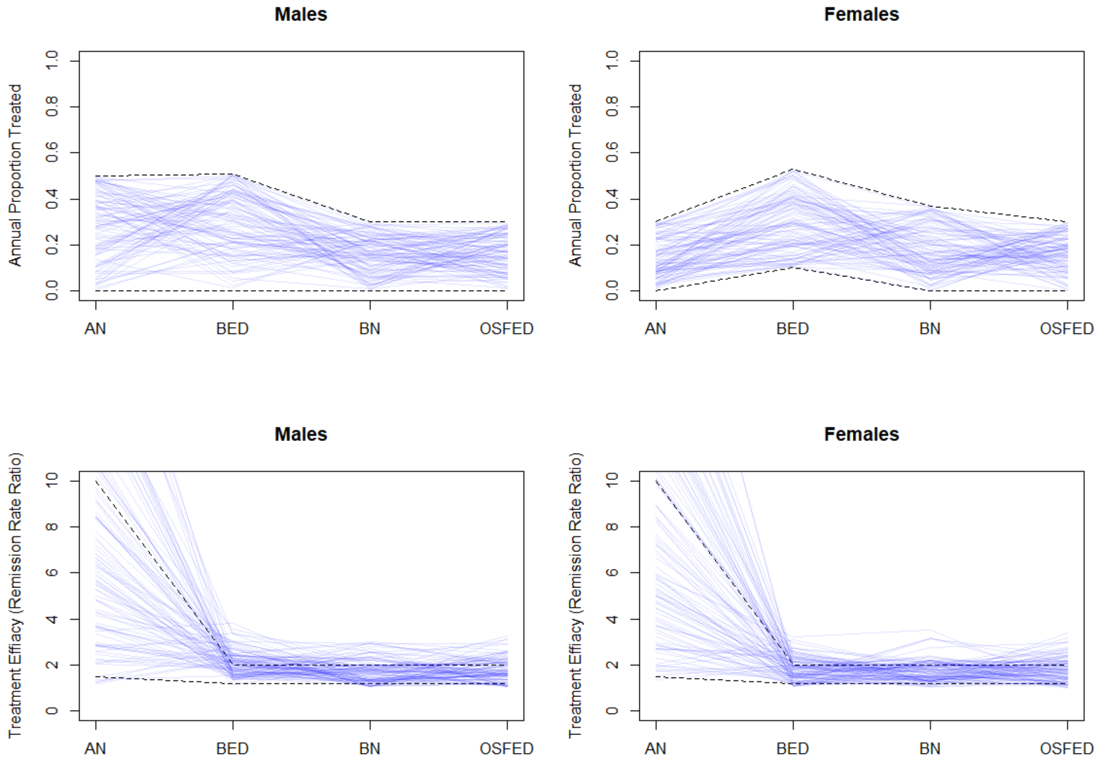

© 2019 Ward ZJ et al. *JAMA Network Open.*

eFigure 9. Interdisorder Transitions

Black lines: Prior Search Bounds, Blue lines: Top 100 Parameter Sets

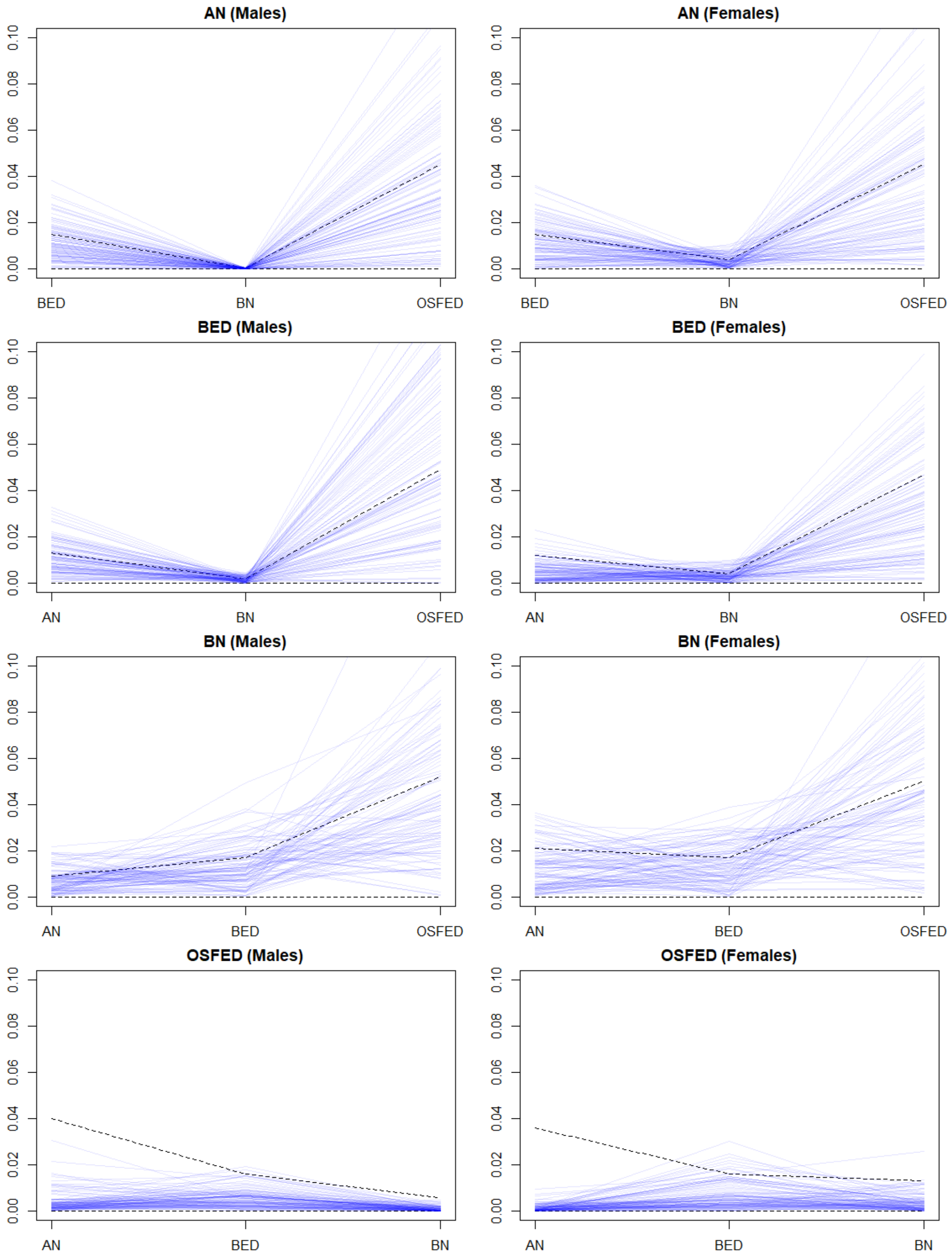

© 2019 Ward ZJ et al. *JAMA Network Open.*

eFigure 10. Mortality

Black lines: Prior Search Bounds, Blue lines: Top 100 Parameter Sets

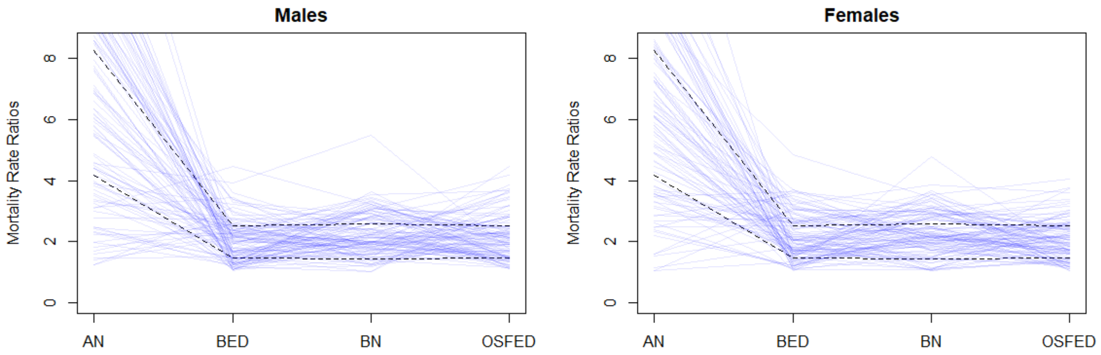

eFigure 11. Annual Prevalence by Age

Shaded areas indicate 95% UIs.

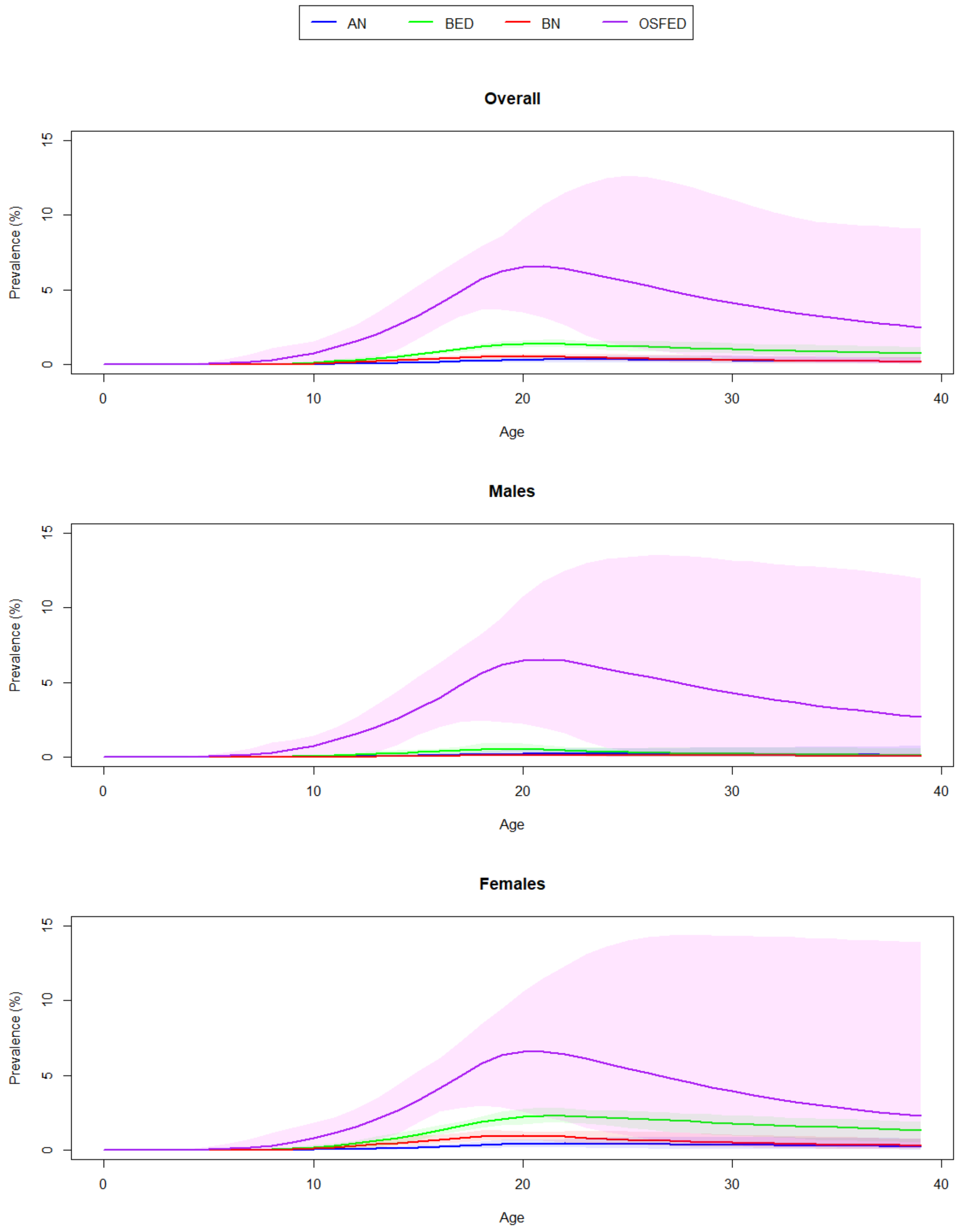

© 2019 Ward ZJ et al. *JAMA Network Open.*
